# Efficacy of the Autoimmune Protocol Diet as Part of a Multi-disciplinary, Supported Lifestyle Intervention for Hashimoto’s Thyroiditis

**DOI:** 10.7759/cureus.4556

**Published:** 2019-04-27

**Authors:** Robert D Abbott, Adam Sadowski, Angela G Alt

**Affiliations:** 1 Independent Researcher, Resilient Roots Functional and Evolutionary Medicine, Charlottesville, USA; 2 Helfgott Research Institute, National University of Natural Medicine, Portland, USA; 3 Independent Researcher, Columbia, USA

**Keywords:** autoimmune thyroid disease, hashimoto's thyroiditis, paleo diet, quality of life, lifestyle, health coaching, nutrition

## Abstract

Background

Hashimoto’s thyroiditis (HT), also known as chronic lymphocytic thyroiditis, is an autoimmune disorder affecting the thyroid gland and is the most common cause of hypothyroidism in the US. Despite medical management with thyroid hormone replacement, many individuals with HT continue to experience symptoms and impaired quality of life. Given the limited number of efficacious treatments outside of hormone replacement and the overall burden of continued symptomatic disease, this pilot study was designed to determine the efficacy of a multi-disciplinary diet and lifestyle intervention for improving the quality of life, clinical symptom burden, and thyroid function in a population of middle-aged women with HT.

Materials and methods

The study recruited 17 normal or overweight (body mass index (BMI) <29.9) female subjects between the ages of 20 and 45 with a prior diagnosis of HT. The 17 women participated in a 10-week online health coaching program focused on the implementation of a phased elimination diet known as the Autoimmune Protocol (AIP). The 36-Item Short Form Health Survey (SF-36) and Cleveland Clinic Center for Functional Medicine’s Medical Symptoms Questionnaire (MSQ) were used to measure the participant’s health-related quality of life (HRQL) and clinical symptom burden, respectively, before and after the 10-week program. The participants completed serologic testing that included a complete blood cell count (CBC) with differential, complete metabolic profile (CMP), thyroid function tests, including thyroid stimulating hormone (TSH), total and free T4, and total and free T3, thyroid antibodies, including thyroid peroxidase antibodies (TPO) and anti-thyroglobulin antibodies (TGA), and high-sensitivity C-reactive protein (hs-CRP).

Results

Sixteen women (n = 16) completed the SF-36 and MSQ before and after the 10-week program. There was a statistically significant improvement in HRQL as measured by all eight subscales of the SF-36 with the most marked improvements noted in the physical role functioning, emotional role functioning, vitality, and general health subscales. The clinical symptom burden, as measured by the MSQ, decreased significantly from an average of 92 (SD 25) prior to the program to 29 (SD 20) after the program. There were no statistically significant changes noted in any measure of thyroid function, including TSH, free and total T4, free and total T3 (n = 12), as well as thyroid antibodies (n = 14). Inflammation, as measured by hs-CRP (n = 14), was noted to significantly decrease by 29% (p = 0.0219) from an average of 1.63 mg/L (SD 1.72) pre-intervention to 1.15 mg/L (SD 1.31) post-intervention.

Conclusions

Our study suggests that an online diet and lifestyle program facilitated by a multi-disciplinary team can significantly improve HRQL and symptom burden in middle-aged female subjects with HT. While there were no statistically significant changes noted in thyroid function or thyroid antibodies, the study’s findings suggest that AIP may decrease systemic inflammation and modulate the immune system as evidenced by a decrease in mean hs-CRP and changes in white blood cell (WBC) counts. Given the improvements seen in the HRQL and participants’ symptom burden as well as markers of immune activity and inflammation, further studies in larger populations implementing AIP as part of a multi-disciplinary diet and lifestyle program are warranted.

## Introduction

Hashimoto’s thyroiditis (HT) is the most common autoimmune thyroid condition and the overall cause of hypothyroidism in the Western world, disproportionately affecting Caucasian females over men and other ethnic groups [1]. HT is a complex disease with multiple etiologic factors, including environmental exposures, drug use, pregnancy, nutritional intake, and infectious diseases [2]. Family and twin-based studies have revealed various genetic susceptibilities primarily related to variations in an individual’s human leukocyte antigen (HLA) genotype as well as variations in numerous cytokines and the vitamin D receptor [2]. Given the complexity of HT, with numerous genetic contributors and our emerging understanding of additional environmental mediators, further research in therapies that can positively modify known environmental factors and mitigate risk for genetically susceptible individuals is warranted.

Currently, for individuals diagnosed with HT, there are few, if any, efficacious treatments outside of thyroid hormone replacement. Winther et al. showed that, in a cohort of 78 consecutive individuals newly diagnosed with HT, baseline markers of quality of life, as measured by the 36-Item Short Form Health Survey (SF-36), were significantly lower than normative healthy controls [3]. Despite slight improvements in thyroid-specific and mental-health-specific quality of life, individuals with HT persisted with overall lower quality of life as compared to healthy controls even after six months of treatment with levothyroxine therapy [3]. Additionally, even after normalizing thyroid function via hormonal replacement, many individuals with HT persist with numerous symptoms, such as chronic fatigue, dry skin, hair loss, chronic irritability, and nervousness, impairing quality of life [4].

To address the need for additional therapeutic options targeted at improving quality of life and symptom burden in individuals with HT, the objective of this study was to determine the efficacy of a multi-week diet and lifestyle intervention implemented by a physician, a team of nutritional therapy practitioners (NTPs), and health coaches. The study authors hypothesized that the multi-dimensional intervention would improve the participant's HRQL as well as decrease the participant's clinical symptom burden. The study authors additionally hypothesized that the intervention would improve thyroid function as measured by a decrease in TSH and increases in free and total T3 and T4. It was speculated that some individuals would require less thyroid replacement medication after the 10-week intervention. Finally, the study authors sought to explore the effect of the 10-week intervention on inflammation and immune activity as measured by high-sensitivity C-reactive protein (hs-CRP), white blood cell (WBC) count, differential cell counts, and thyroid antibodies, including thyroid peroxidase (TPO) antibodies and anti-thyroglobulin antibodies (TGA).

There have been numerous clinical trials evaluating the use of dietary interventions for a variety of autoimmune diseases, including inflammatory bowel disease (IBD), multiple sclerosis, psoriasis, celiac disease, autoimmune thyroiditis, and rheumatoid arthritis [5-11]. A recent 2017 review assessed the role of iodine, selenium, vitamin D, and gluten on the management of patients with HT [10]. The authors concluded that the role of a gluten-free diet may be of benefit for patients with HT independent of a comorbid diagnosis of celiac disease. Despite the potential benefit from this dietary elimination, they speculated, however, that quality of life could be negatively impacted given the restrictive nature of the gluten-free diet.

Outside of our current understanding regarding the importance of certain key nutrients for the optimal functioning of the thyroid gland, namely iodine, selenium, zinc, iron, B12, and lipid soluble vitamins, including A, E, D, and K, there are no specific dietary guidelines or recommendations for individuals with autoimmune thyroid disease. While some have speculated that over-consumption of dietary goitrogens could negatively impact thyroid functioning, there is an absence of rigorous trials suggesting the negative effects of such foods when consumed in normal proportions.

In seeking to identify a dietary template and feasible lifestyle intervention that could positively improve HRQL and symptom burden in individuals with HT, five criteria were outlined: (1) Consumption of foods high in micronutrients containing, but not limited to, the aforementioned nutrients essential for thyroid functioning; (2) Elimination of foods with low nutritional value (sugar-sweetened beverages, ultra-processed foods, etc.) and foods that could result in an aberrant immune response via dysregulated antigen presentation or detrimentally affect both the gut microbiome and the integrity of the gastrointestinal barrier; (3) Implementation of dietary changes in phases, utilizing education and support from health coaches, NTPs, and a physician to improve dietary adherence; (4) Facilitation of the dietary and lifestyle intervention as part of an online community allowing participants to engage with other study members; (5) Prior clinical evidence for the efficacy of the intervention for a specific autoimmune disease.

In light of these five criteria, the Autoimmune Protocol (AIP), as implemented within the “SAD (Standard American Diet) to AIP in SIX” online, community-based health coaching program was the dietary and lifestyle program identified as most likely to result in participant adherence and symptom improvement. AIP, as implemented by the “SAD to AIP in SIX” program, was previously studied in individuals with inflammatory bowel disease (IBD) and was shown to be able to induce remission and improve symptoms in over 70% of participants [5].

In terms of its dietary composition, AIP is a modification of the Paleolithic diet that begins with an elimination of specific foods, dietary additives, emulsifiers, and western dietary patterns that have been implicated in disrupting the flora of the gastrointestinal microbiome as well as the intestinal barrier, leading to dysregulated antigen presentation and the development of autoimmunity [12-17]. In addition to the eliminated foods, AIP additionally promotes the consumption of nutrient-dense whole foods such as vegetables, fruits, mono and polyunsaturated fatty acids, tubers, wild game, poultry, organ meats, and non-processed meats.

## Materials and methods

Study design and measures

Prior to enrolling in the trial, advertising for the study was completed across various social media outlets and local practitioners treating patients with HT. Upon receiving correspondence from interested participants, communication was initiated by study investigators to assess the participants’ ability to enroll in the intervention. A total of 456 subjects were screened with inclusion and exclusion criteria, resulting in 17 subjects eligible for inclusion in the trial. Inclusion criteria consisted of English-speaking male or females, 20-45 years of age, with a diagnosis of HT and a body mass index (BMI) between 18.4 and 29.9. Exclusion criteria consisted of individuals outside the listed age or BMI criteria, no definitive diagnosis of HT, prior experience with AIP for >30 days, pregnant, breastfeeding, six months postpartum, presence of other comorbidities, including hypertension, diabetes, heart disease, heart failure, liver failure, chronic or end-stage kidney disease, use of medications outside of Food and Drug Administration (FDA)-approved thyroid replacement medications, or an individual being unable to complete a two-week washout period before the start of the trial. In selecting the inclusion and exclusion criteria, the study authors sought to identify normal or overweight (non-obese) premenopausal women to minimize the influence of hormonal variations between pre and post-menopausal women as well as to minimize the likelihood for rapid weight loss in obese individuals. The study authors sought to minimize the risk of adverse effects, complications, and variations in thyroid function secondary to other disease processes by excluding individuals with chronic organ disease/organ failure as well as pregnant or breastfeeding women and women in the early post-partum period.

The two-week washout period consisted of a screening visit prior to the initiation of the formal dietary intervention where subjects signed informed consent, provided demographic information, completed study questionnaires, including a validated quality of life survey, the 36-Item Short Form Health Survey (SF-36), the Cleveland Clinic Center for Functional Medicine’s Medical Symptom Questionnaire (MSQ), and the National Institutes of Health’s (NIH's) food frequency questionnaire (FFQ) [18-19]. Participants provided fasting blood samples, including complete blood cell count with differential (CBC), complete metabolic profile (CMP), thyroid stimulating hormone (TSH), free T4, free T3, total T4, total T3, reverse T3, thyroid peroxidase (TPO) antibodies, anti-thyroglobulin antibodies (TGA), 25-hydroxycholecalciferol, and high sensitivity C-reactive protein ( hs-CRP). In addition, Genova Diagnostics Laboratory supplied organic acid tests (NutrEval FMV™, Genova Diagnostics Laboratory, NC, USA) as well as comprehensive stool analysis (GI Effects™, Genova Diagnostics Laboratory, NC, USA) for participants to complete during the washout period.

After completion of the washout period, participants began a 10-week online dietary and lifestyle intervention, which consisted of a six-week process of food elimination, the addition of nutrient-dense foods, and a focus on lifestyle modifications, followed by a four-week maintenance phase (during which no food group reintroductions were allowed), using the “SAD (Standard American Diet) to AIP in SIX” group health coaching program. Food eliminations, additions, and lifestyle modifications were done in weekly stages. Foods eliminated included all grains, legumes, nightshades, dairy, eggs, coffee, alcohol, nuts, seeds, refined/ultra-processed sugars, oils, and food additives. Micronutrient-dense food additions included foods rich in mono and polyunsaturated fatty acids, bone broth, seafood, fermented foods, and organ meats. Lifestyle modifications included the promotion of support systems, sleep hygiene, stress management, movement, and increasing time spent outdoors.

Certified health coaches and NTPs led the dietary and lifestyle intervention, educating participants with reasons for food eliminations, additions, and particular lifestyle modifications. They provided plans to help participants sustain the rigorous elimination process such as menu planning, grocery shopping, cooking foods, and recipe guides. All of the education and support was provided virtually via email and a private Facebook group accessible only to invited members. The health coaches and NTPs led daily discussions on the changes participants were implementing, provided encouragement when participants faced challenges, answered questions regarding the study process, and troubleshot with participants who experienced difficulty with the protocol.

For the purposes of the study, the health coaches and NTPs also collaborated with the lead physician in the event of any medical concerns for study participants. Through lab testing, FFQ, MSQ, and SF-36, the physician was able to recognize specific issues that individual participants were experiencing and met with health coaches to discuss methods of addressing the issues within the study framework. Where appropriate, the lead health coach and physician discussed with individual participants regarding any concerns and helped the participant address them effectively.

At the end of the intervention, questionnaires and all laboratory work, including organic acid and stool samples, were repeated. The study was conducted in full accordance with the Valley Health Research Policies and Procedures and all applicable Federal and State laws and regulations, including 45 CFR 46, 21 CFR parts 50, 54, 312, 314, and 812, as well as the Good Clinical Practice: Consolidated Guideline approved by the International Conference on Harmonisation. Participants were allowed to drop out of the study at any time.

Data collection, analysis, and outcomes

A per-protocol analysis was conducted using data from participants completing the study in its entirety (n = 16). Individuals (n = 2) who decreased thyroid medications during the study were not included in the final group analyses of thyroid hormone parameters but were included in the analyses of thyroid antibodies, including TPO antibodies and TGA. Individuals (n = 2) who were acutely ill during either the pre-intervention or the post-intervention laboratory testing were not included in the final group analyses of thyroid hormone parameters, thyroid antibodies, hs-CRP, white blood cell (WBC) count, or differential cell count analysis. Data from all 17 participants completing pre-intervention testing and 16 participants completing post-intervention testing are included in the Appendix. Specific denotations are listed in the Appendix to designate the specific data described above that was not included in the respective per protocol analyses as well as significant outlying data that was not included in the post-hoc secondary analyses.

Paired t-tests were calculated for all SF-36, MSQ, thyroid parameters, including antibodies, WBC count, differential cell counts, hs-CRP, self-reported weight, and BMI results from pre- to post-dietary intervention using Prism 8 (GraphPad Software, CA, US), resulting in a total of 27 tested parameters. It was noted during initial statistical calculations that several individual subscales of the SF-36 failed the Shapiro Wilk test for normality, and thus, all SF-36 data sets could not be assumed to be normally distributed. Wilcoxon Signed-Rank tests were thus performed for all eight subscales of the SF-36, and the respective median values were calculated and recorded alongside the respective inter-quartile range (IQR). All other data sets were assumed to be normally distributed, with statistics from the resulting paired t-tests represented as a mean (M) and standard deviation (SD). Effect sizes for normally distributed samples were also calculated using Hedge's g statistic (g) and are listed when appropriate.

In order to correct for error when performing statistical analyses for multiple hypotheses, balancing the risk of creating both Type I and Type II errors, the study authors utilized a false discovery rate control adjustment outlined by Glickman, Rao, and Schultz with a maximum false discovery rate d = 0.05 for n = 27 statistical tests. As part of this adjustment, new thresholds for statistical significance were set and are listed with their originally calculated and corresponding p-value in the Appendix [20].

The study's primary outcome was a significant change in SF-36 measures. The study's secondary outcomes consisted of changes in clinical symptom burden as measured by the MSQ, changes in thyroid parameters, including thyroid antibodies, changes in WBC and differential cell counts, and changes in hs-CRP. Measures from the organic acid and stool testing were exploratory in nature, however, pre-intervention data from these tests were utilized to inform specific dietary recommendations for individuals during week five of the intervention. These recommendations varied and were based on aspects of the organic acid test suggesting deficits in B vitamins or minerals such as magnesium, copper, riboflavin, B6, folate, or B12 as well as aspects of the stool testing suggesting overgrowth of bacterial organisms, fat malabsorption, or pancreatic insufficiency. Clinically relevant specifics of the stool and organic acid testing from individuals pre and post-intervention, as well as clinical recommendations provided midway through the 10-week intervention, are discussed as part of participant case summaries in the Appendix. Adverse effects were monitored throughout the study and recorded.

## Results

Seventeen women meeting the study’s inclusion and exclusion criteria were enrolled and completed the two-week washout period. Baseline demographics, including age, height, weight, BMI, and ethnicity, are listed in Table 1. Fifteen out of 17 (87.5%) of the women were noted to be Caucasian. One participant became pregnant during the study and, as a result, discontinued participation in the study and was not included in the final analysis.

**Table 1:** Baseline demographics of participants included for final analysis y (years), in (inches), lbs (pounds), BMI (body mass index), SD (standard deviation), N (sample size)

Variable	N	Mean (SD)
Age, y	16	35.6(5.7)
Height (in)	16	65.3(2.4)
Weight (lbs)	16	149.5(19.5)
BMI	16	24.9(2.6)

Sixteen women (n = 16) completed the SF-36 and MSQ before and after the 10-week program. There was a statistically significant improvement in HRQL as measured by all eight subscales of the SF-36 (Table 2) with the most marked improvements noted in the physical role functioning subscale with a pre-intervention median = 25, IQR 88, and post-intervention median = 100, IQR 50 (p = 0.001), the vitality subscale with a pre-intervention median = 23, IQR 19, and post-intervention median = 58, IQR 34, p < 0.0001, and the general health subscale with a pre-intervention median = 40, IQR 26, and post-intervention median = 70, IQR 35 (p < 0.0001)

**Table 2:** SF-36 paired t-tests results and statistics SF-36 (36-Item Short Form Health Survey), Pre (pre-intervention), Post (post-intervention), N (sample size), IQR (inter-quartile range), P (p value), (*) denotes statistically significant p value

	SF-36 Physical Functioning	SF-36 Physical Role Functioning	SF-36 Emotional Role Functioning	SF-36 Vitality	SF-36 Mental Health	SF-36 Social Role Functioning	SF-36 Bodily Pain	SF-36 General Health
N	16	16	16	16	16	16	16	16
Median (IQR) Pre	80 (29)	25 (88)	33 (92)	23 (19)	54 (25)	63 (22)	68 (22)	40 (26)
Median (IQR) Post	95 (10)	100 (50)	78 (19)	58 (34)	78 (19)	81 (22)	78 (21)	70 (35)
Median of Differences (IQR)	10 (10)	50 (75)	41 (67)	33 (29)	22 (12)	19 (37)	23 (32)	28 (21)
P	0.0001*	0.001*	0.0063*	<0.0001*	<0.0001*	0.0057*	0.0112*	<0.0001*

**Figure 1:**
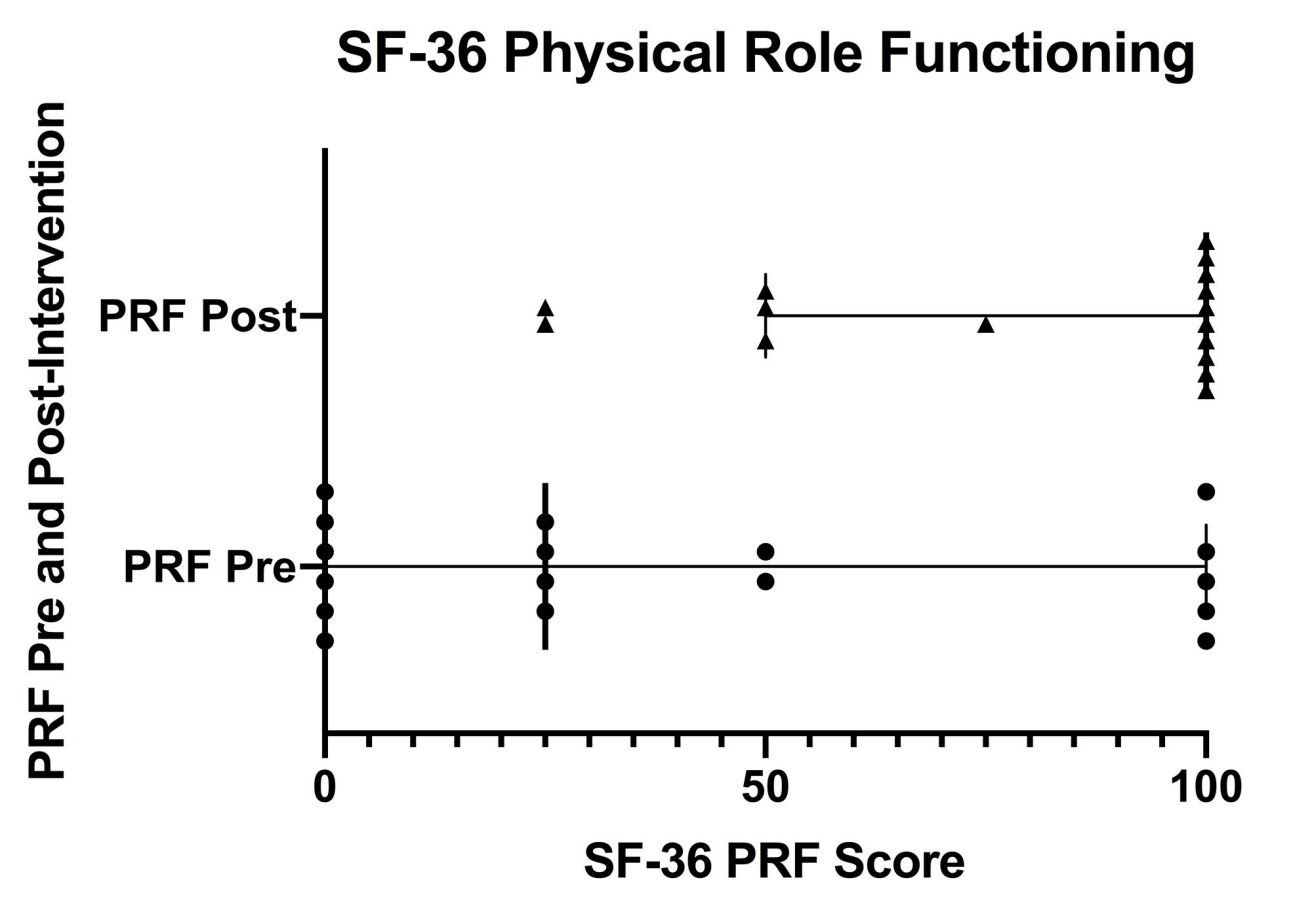
SF-36 physical role functioning scores pre- and post-intervention SF-36 (36-Item Short Form Health Survey), PRF (physical role functioning), Pre (pre-intervention), Post (post-intervention)

Figure 1 displays a scatter plot of SF-36 Physical Role Functioning scores pre- and post-intervention. Individual pre-intervention scores are depicted with circles and individual post-intervention scores are depicted with triangles. Error bars indicate the inter-quartile range (IQR). Solid, bolded vertical lines within the IQR indicate the median.

**Figure 2:**
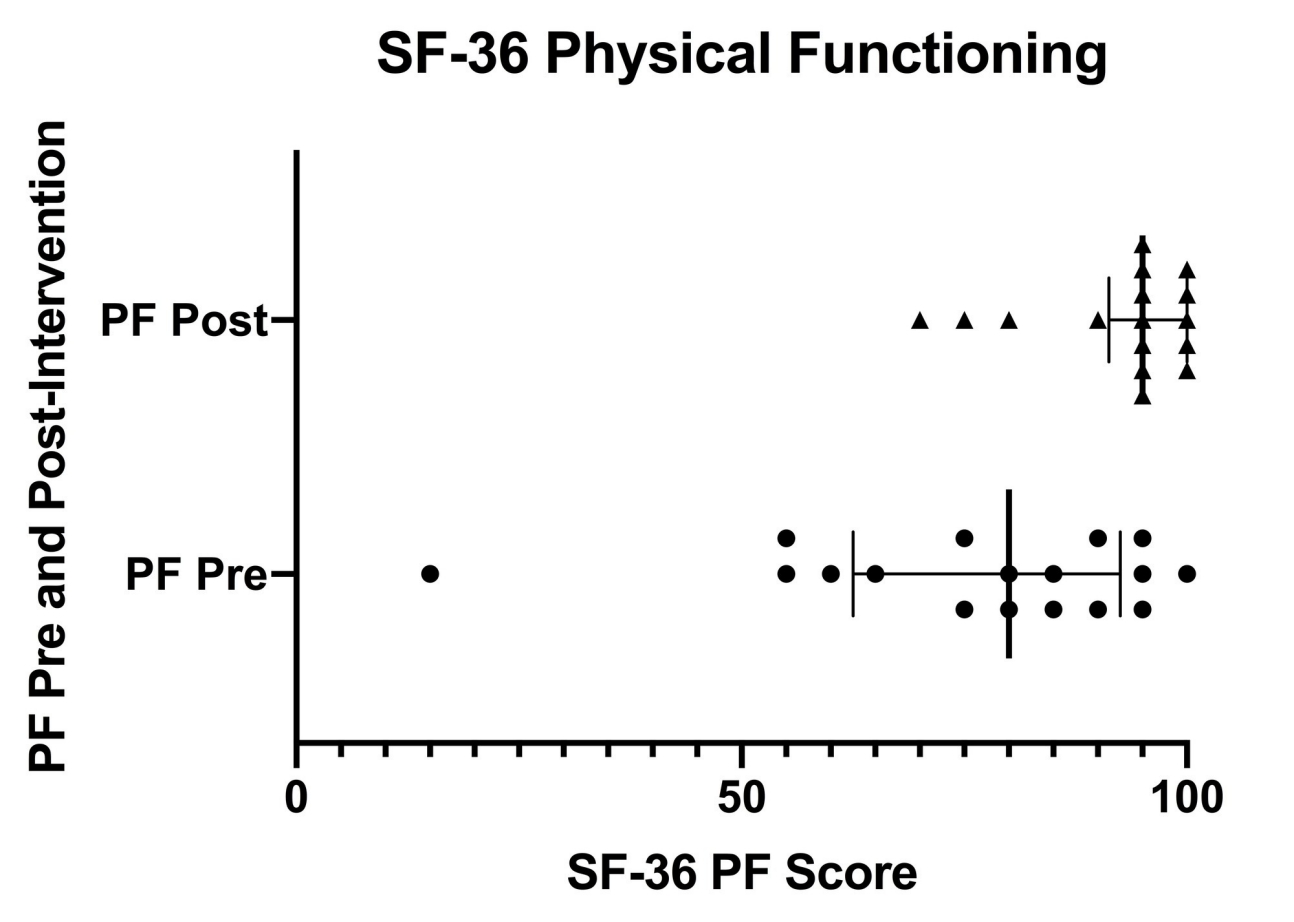
SF-36 physical functioning scores pre- and post-intervention SF-36 (36-Item Short Form Health Survey), PF (physical functioning), Pre (pre-intervention), Post (post-intervention)

Figure 2 displays a scatter plot of SF-36 physical functioning scores pre- and post-intervention. Individual pre-intervention scores are depicted with circles and individual post-intervention scores are depicted with triangles. Error bars indicate the inter-quartile range (IQR). Solid, bolded vertical lines within the IQR indicate the median.

**Figure 3:**
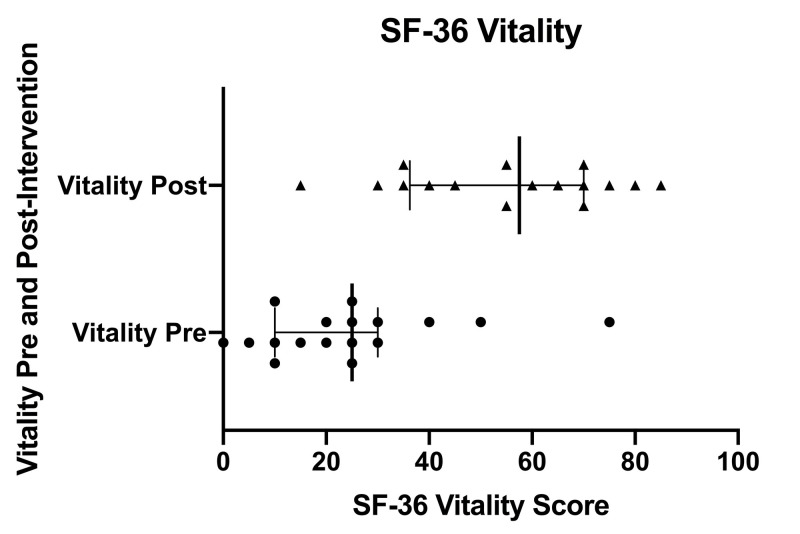
SF-36 vitality scores pre- and post-intervention SF-36 (36-Item Short Form Health Survey), Pre (pre-intervention), Post (post-intervention)

Figure 3 displays a scatter plot of SF-36 vitality scores pre- and post-intervention. Individual pre-intervention scores are depicted with circles and individual post-intervention scores are depicted with triangles. Error bars indicate the inter-quartile range (IQR). Solid, bolded vertical lines within the IQR indicate the median.

**Figure 4:**
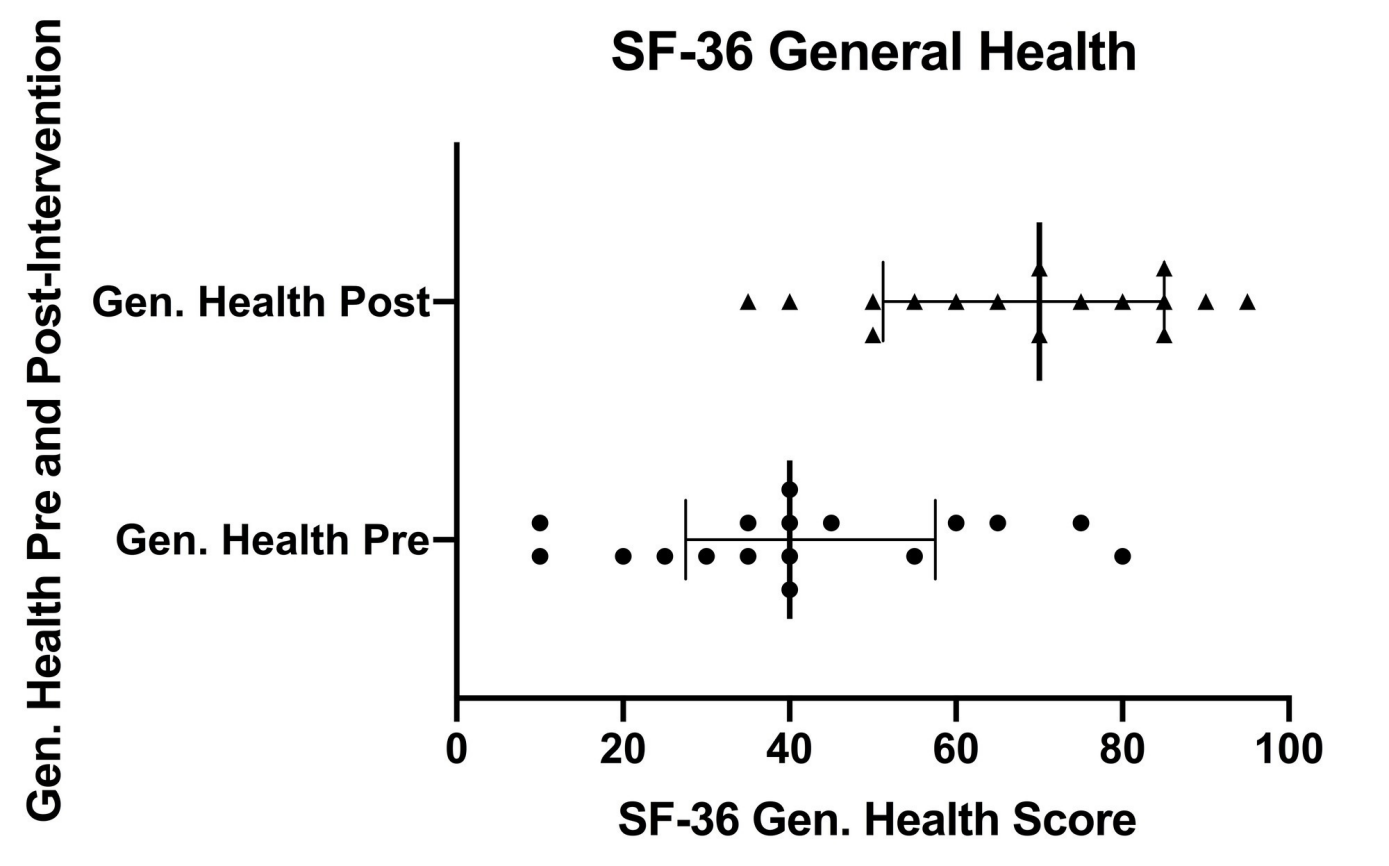
SF-36 general health scores pre- and post-intervention SF-36 (36-Item Short Form Health Survey), Gen. Health (general health), Pre (pre-intervention), Post (post-intervention)

Figure 4 displays a scatter plot of SF-36 general health scores pre- and post-intervention. Individual pre-intervention scores are depicted with circles and individual post-intervention scores are depicted with triangles. Error bars indicate the inter-quartile range (IQR). Solid, bolded vertical lines within the IQR indicate the median.

**Figure 5:**
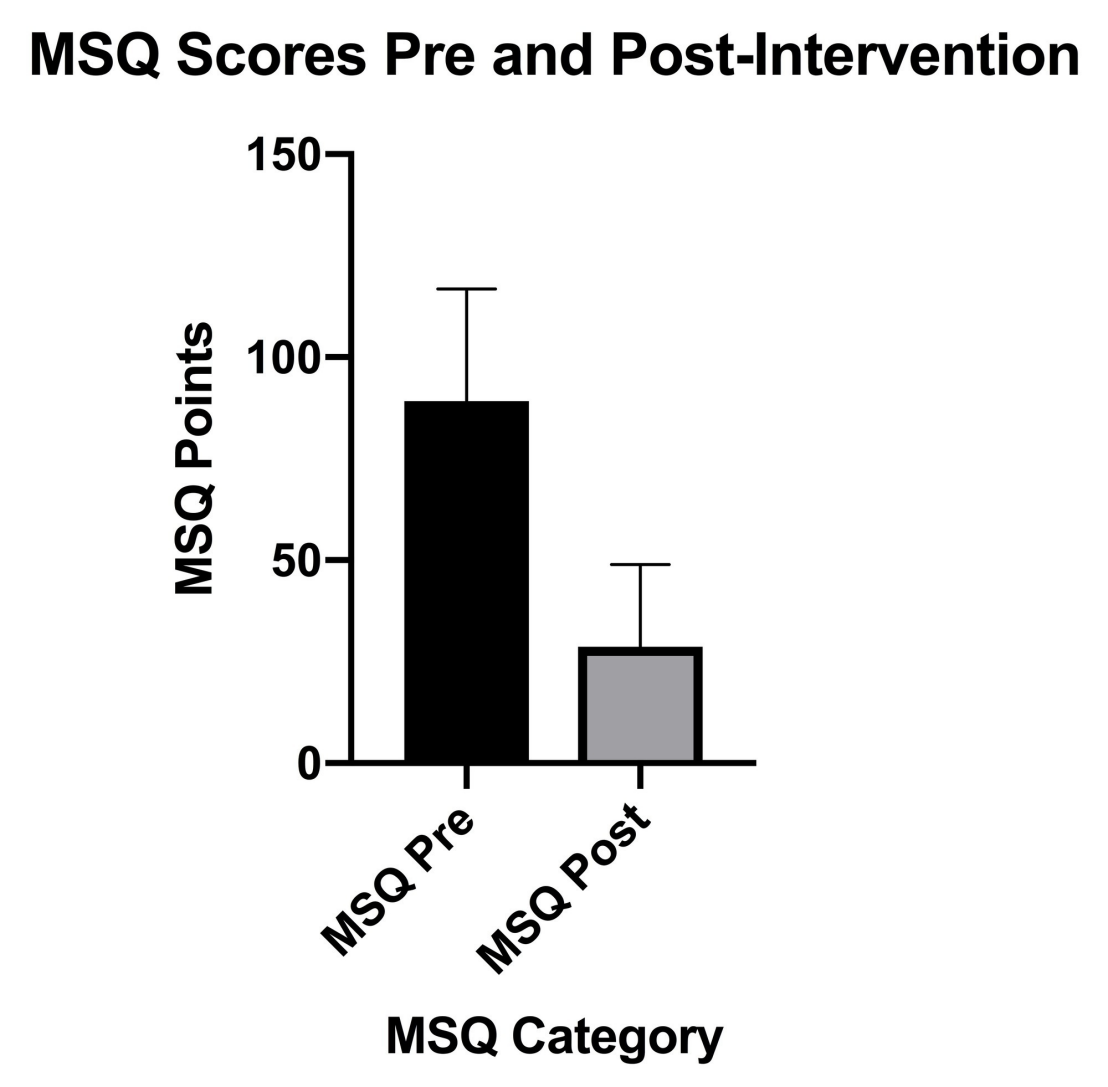
MSQ scores pre-intervention to post-intervention MSQ (Medical Symptoms Questionnaire), Pre (pre-intervention), Post (post-intervention), SD (standard deviation), error bars indicate SD

The clinical symptom burden as determined by MSQ (Figure 5), which measures symptoms over a four-week period, decreased significantly from pre-intervention (M = 92, SD 25) to post-intervention (M = 29, SD 20), n = 16, t(15) = 9.3, p < 0.0001 with a large effect size (g = 2.81).

**Figure 6:**
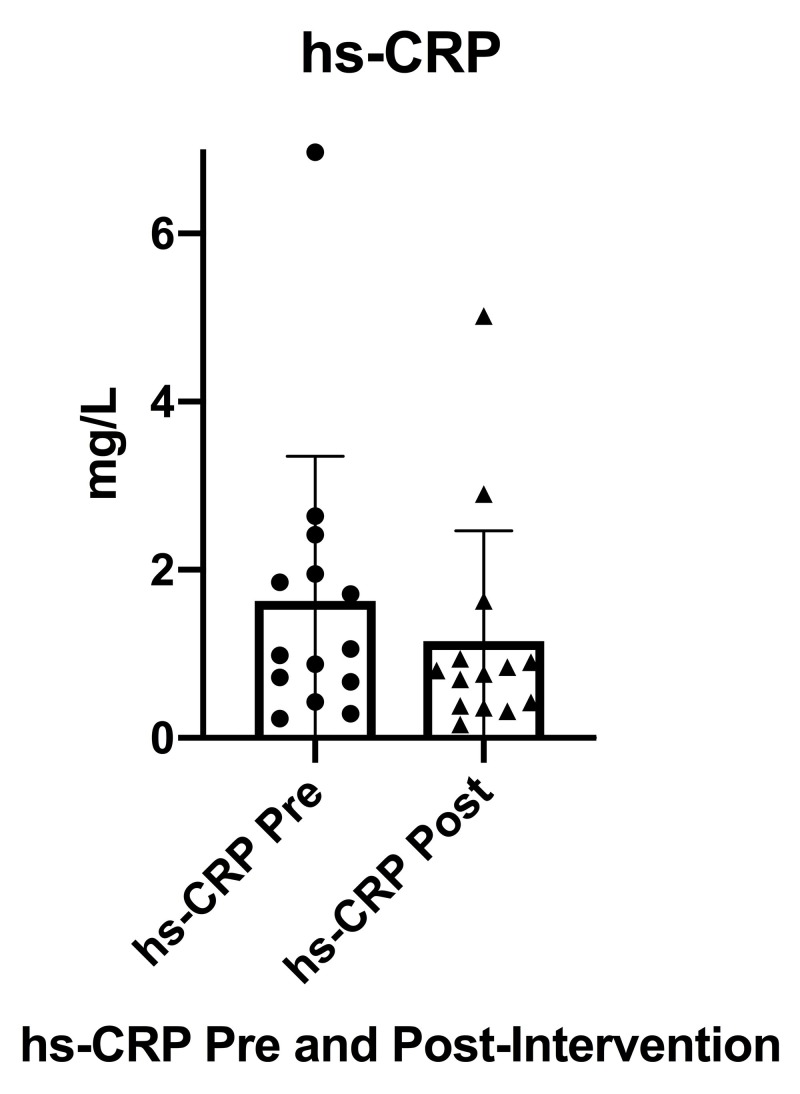
Serum hs-CRP from pre-intervention to post-intervention hs-CRP (high sensitivity C-reactive protein), Pre (pre-intervention), Post (post-intervention), SD (standard deviation), error bars indicate SD

Inflammation, as measured by hs-CRP (Figure 6), decreased significantly from pre-intervention (M = 1.63 mg/L, SD 1.72) to post-intervention (M = 1.15 mg/L, SD 1.31), n = 14, t(13) = 2.60, p = 0.0219 with a small effect size (g = 0.302). As previously noted, data from two participants who were acutely sick during either the pre- or post-intervention blood chemistry testing were not included in the final analysis for hs-CRP.

It was additionally noted when performing the statistical analysis that one participant had a significantly elevated hs-CRP both pre-and post-intervention when compared to the pre- and post-intervention group means, however, she was not acutely sick during either the pre- or post-intervention blood chemistry testing. While the participant’s hs-CRP was noted to decrease from pre- to post-intervention, her data still remained a significant outlier from the group mean as seen in the previous scatter plot (Figure 6). A post-hoc secondary analysis was conducted removing the statistical outlier, resulting in a sample size of n = 13, a pre-intervention mean, M = 1.22 mg/L, SD 0.81, and post-intervention mean, M = 0.85 mg/L, SD 0.72, t(12) = 2.34, p = 0.037 with a moderate effect size (g = 0.473). The pre- and post-intervention hs-CRP data tables in the Appendix denote the specific data from the two acutely sick individuals described above that was not included in the final analysis as well as the data from the outlier that was not included in the post-hoc secondary analysis.

Pre- and post-statistics for all thyroid markers, including antibodies, are listed in Table 3. Individuals who decreased medication use following initial laboratory testing or during the course of the study (n = 2), as described previously in the methods, were not included in the final analysis. Additionally, data from the two participants (n = 2) who were acutely sick during the pre- or post-intervention thyroid testing were not included in the final analysis for TSH, free T4 and T3, total T3 and T4, and reverse T3. Data regarding antibody levels, however, for these two participants were included in the final data analysis. This resulted in a total of 12 participants analyzed for thyroid markers and 14 analyzed for thyroid antibodies. All data for the 17 participants completing pre-intervention thyroid testing as well as the 16 participants completing post-intervention thyroid testing is listed in the Appendix with specific denotations for the individual data described above that was not included in the final data analysis.

**Table 3:** Thyroid hormone and antibody values pre- and post-intervention with paired t-test statistics TPO (thyroid peroxidase antibodies), TGA (anti-thyroglobulin antibodies), pre (pre-intervention), post (post-intervention), N (sample size), SD (standard deviation), t (t-test statistic), P (p-value), g (Hedges' g)

	TSH (μIU/mL)	Total T3 (ng/dL)	Free T3 (pg/mL)	Reverse T3 (ng/dL)	Total T4 (μg/dL)	Free T4 (ng/dL)	TPO (IU/mL)	TGA (IU/mL)
N	12	12	12	12	12	12	14	14
Mean (SD) pre	2.02(1.46)	97.3(18.0)	2.4(0.6)	17.4(4.3)	7.0(1.1)	1.3(0.4)	225(178)	110(261)
Mean (SD) post	1.98(1.44)	89.0(9.0)	2.4(0.5)	19.1 (5.3)	7.1(1.4)	1.4(0.4)	219(186)	124(293)
t	0.075	1.668	0.1515	1.9717	0.5932	0.841	0.7703	1.4292
P	0.942	0.124	0.882	0.0743	0.565	0.418	0.455	0.176
g	0.029	0.584	0.029	0.355	0.124	0.099	0.035	0.0532

No clinically nor statistically significant changes were seen in TSH, total T3 or T4, and free T3 or T4. Additionally, no clinically nor statistically significant changes were noted for either TPO antibodies or TGA.

White blood cell (WBC) and differential cell counts pre- and post-intervention are listed in Table 4. It was noted that there was a decrease in mean WBC count from a pre-intervention mean of 5.6 x 10^3^ / μL (SD 1.4) to a post-intervention mean of 5.1 x 10^3^/μL (SD 1.4) that did not reach statistical significance, p = 0.1396. As previously noted, two out of the 16 individuals completing the pre- and post-intervention blood chemistry were noted to be acutely sick during either the pre- or post-intervention laboratory testing period and could not be included in the final analysis.

**Table 4:** WBC and differential cell counts pre- and post-intervention with paired t-test statistics WBC (white blood cell), Pre (pre-intervention), Post (post-intervention), N (sample size), SD (standard deviation), t (t-test statistic), P (p-value), g (Hedges' g)

	WBC (10^3^/μL)	Neutrophils (%)	Lymphocytes (%)	Monocytes (%)	Eosinophils (%)
N	14	14	14	14	14
Mean (SD) Pre	5.6(1.4)	57.9(6.6)	30.8(5.3)	8.4(2.3)	2.4(1.6)
Mean (SD) Post	5.1(1.4)	55.9(8.2)	34.0(7.3)	7.4(1.3)	2.1(1.4)
P	0.1396	0.183	0.0286	0.0684	0.385
g	0.311	0.268	0.502	0.535	0.199

**Figure 7:**
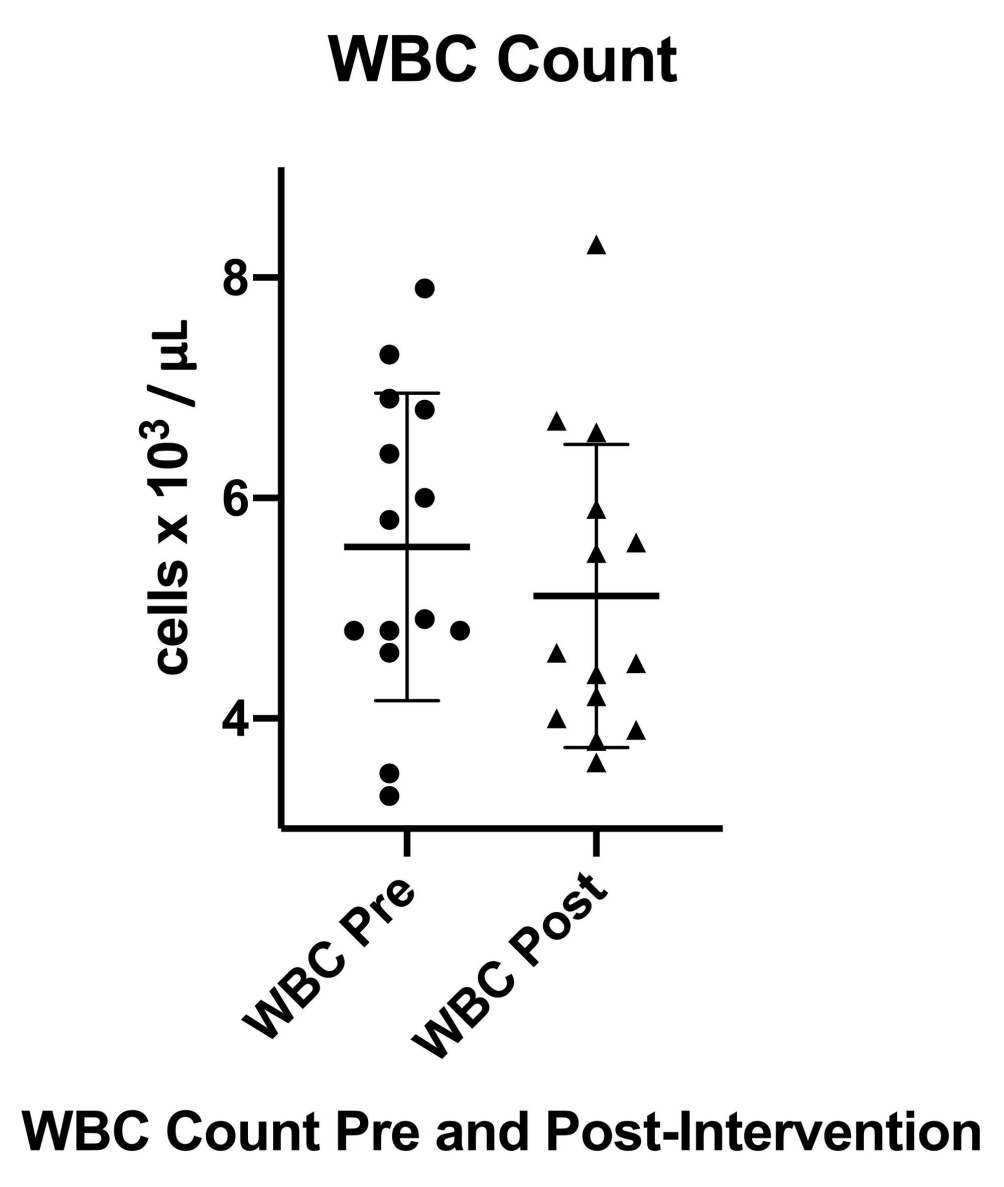
WBC count pre- and post-intervention WBC (white blood cell), Pre (pre-intervention), Post (post-intervention), error bars indicate SD

Figure 7 displays a box plot depicting WBC counts both pre- and post-intervention as well as the mean WBC count with SD. It was noted when performing the paired t-test statistics and creating the box plot that one individual was a significant outlier when compared to the group mean difference in WBC count with an increase in WBC from 6.3 x 10^3^/μL pre-intervention to 8.4 x 10^3^/μL post-intervention. The box plot also depicts two participants who began the intervention with low or borderline low WBC counts (normal > 3.3 x 10^3^/μL) and had increases in WBC count at post-intervention trending toward the group post-intervention mean.

Statistically significant changes were seen in weight (M = 143.4 lbs, SD 16.7, p = 0.002) and BMI (M = 23.9, SD 2.2, p = 0.002) from baseline to post-intervention (Table 5).

**Table 5:** Weight and BMI of all participants pre- and post-intervention, (*) denotes statistically significant P value (p < 0.05) BMI (body mass index), lbs (pounds), N (sample size), SD (standard deviation), Pre (pre-intervention), Post (post-intervention), P (p-value)

	Weight (lbs)	BMI
N	16	16
Mean (SD) Pre	149.5(19.5)	24.9(2.6)
Mean (SD) Post	143.4(16.7)	23.9(2.2)
P	0.002*	0.002*

These results remained significant when a subgroup analysis was performed on participants with a baseline BMI ≥ 25 (M = 27.1, SD 1.0) to post intervention (M = 25.8, SD 1.0, p = 0.011) (Table 6).

**Table 6:** Weight and BMI of overweight subjects (24.9 < BMI < 29.9) pre- and post-intervention, (*) denotes statistically significant P value (p < 0.05) BMI (body mass index), lbs (pounds), N (sample size), SD (standard deviation), Pre (pre-intervention), Post (post-intervention), P (p-value)

	Weight (lbs)	BMI
N	8	8
Mean (SD) Pre	163.0(19.1)	27.1(1.0)
Mean (SD) Post	155.1(15.3)	25.8(1.0)
P	0.011*	0.011*

It should be noted that the review of the individual FFQs was used to determine qualitative and compositional changes in dietary habits with regards to eliminated foods as part of AIP and was not used to determine portion sizes or total caloric intake.

Six out of the 13 women beginning the study on thyroid replacement medication decreased their dose of hormone replacement medication after the 10-week intervention. All three individuals who decreased the dosages of their medication following the pre-intervention testing made subsequent decreases in their medication dosage in addition to three individuals who decreased their medication dosages following post-intervention laboratory testing. All three of the women who began the study without the use of hormone replacement medication continued without the use of replacement medication as of the final post-intervention study visit.

There were no moderate to severe adverse effects noted during the duration of the study. Some study participants reported mental challenges during the initial phases of the dietary eliminations, however, this is was offset very quickly by decreases in overall symptom burden.

## Discussion

This single-arm pilot study adds to the current evidence that AIP, a modification of the Paleolithic diet involving the elimination and promotion of certain foods, may help alleviate symptoms and improve quality of life in participants with an autoimmune disease. We demonstrated preliminary efficacy in participants with HT, via statistically and clinically significant improvements in SF-36 and MSQ scores, as well as statistically and clinically significant decreases in hs-CRP, weight, and BMI despite no statistically significant changes in thyroid laboratory markers or thyroid antibodies. Reviews of FFQs from participants during the 10-week program revealed 95%-100% adherence to the strict elimination criteria. The strict dietary adherence is most likely a result of the intensive health coaching and community-based structure providing both education and a source of communal accountability. Preliminary study questionnaires revealed a majority of the participants reporting familiarity with AIP. Some participants even reported previous attempts at the AIP dietary protocol for fewer than five days, given the lack of education about the dietary approach, support services, and communal accountability as well as the overall challenge in preparing 100% AIP-compliant meals. The role of the physician, health coaches, and NTPs, as well as the participants’ communal group environment, cannot be understated and appears to be the primary mediating elements behind the high rate of adherence.

These results additionally suggest that the AIP diet and concomitant lifestyle modification, as implemented by a multi-disciplinary team, can be safely used as adjunctive treatments for people with HT who are already utilizing hormone replacement therapy. There were no reported serious adverse effects, with many participants actually reporting noticeable positive changes within the first four weeks of the elimination diet. While there were no observed changes in mean thyroid laboratory markers and antibodies, six out of 13 women (46.1%) who were taking thyroid replacement medication at the beginning of the study actually decreased their dosage of hormone replacement medication by the end of the 10-week study period. All three women who were asked to decrease or alter their medication dosing at the beginning of the study due to pre-intervention laboratory findings of low TSH or abnormal free hormone levels actually found they needed to decrease their medications even further following the 10-week program. Three women who began the study without the utilization of hormone replacement medication were able to continue without hormone replacement medication. One individual who enrolled with subclinical hypothyroidism and elevated thyroid antibodies diagnostic of HT had a significantly higher post-intervention TSH, yet nearly identical free and total hormone levels as well as lower TPO antibodies at post-intervention. It is difficult to predict the continued disease course of this specific individual outside of the study structure, however, it is likely that she would require both hormonal replacement therapy with concomitant dietary and lifestyle support to manage any further progression of autoimmune thyroiditis.

Despite the lack of a significant clinical change in mean thyroid markers, including antibodies, the statistically and clinically significant decreases observed for hs-CRP point to modulation of the overall immune and inflammatory response underlying autoimmune thyroiditis. Additionally, a secondary post-hoc analysis of changes in WBC count from pre- to post-intervention (n = 13) that removed one statistical outlier resulted in a statistically significant change in mean WBC count from 5.5 x 10^3^/μL (SD 1.4) to 4.9 x 10^3^/μL (SD 1.1), p = 0.0205. There was also a noted increase in mean lymphocyte count from pre- to post-intervention (p = 0.0286) that could not be assumed to be statistically significant after correcting for multiple hypothesis testing using the false discovery rate correction. It is clear from these statistical examinations that there is some underlying modulation of the immune system that is not as statistically or clinically robust as the changes in HRQL and clinical symptom burden but, nonetheless, should be acknowledged and explored in further study of the AIP dietary intervention. It is also interesting to note that the pre-intervention mean hs-CRP in the post-hoc secondary analysis that included n = 13 subjects was classified as intermediate risk while the post-intervention mean actually dropped below 1.0 mg/L into low-risk categorization.

The authors speculate that it is possible that one would observe an eventual decrease in thyroid antibodies and a decreased need for supplemental medication as well as more robust changes in immune and inflammatory markers in participants adhering to the AIP dietary principles for additional periods of six to 12 months.

In speculating as to the mechanisms behind the observed positive changes in quality of life, symptom burden, as well as hs-CRP, we suggest a further examination of the original criteria set forth for a feasible and efficacious dietary and lifestyle intervention. Self-reported FFQs and dietary journals provided throughout the duration of the study indicate the inclusion of foods with greater nutrient density by all participants and the exclusion of less nutrient-dense foods. Qualitative post-intervention surveys additionally appear to indicate that the study participants received a positive benefit from the gradual nature of the dietary eliminations, the consistent support from the multi-disciplinary team, and the ability to interact with other participants making the same dietary and lifestyle changes. There was a statistically and clinically significant change in weight and BMI from pre- to post-intervention within both the cohort as well as a smaller overweight subpopulation. Despite the dietary intervention lacking a focus on caloric quantification, the restriction of specific macronutrients, such as carbohydrates or fats, or an emphasis on the promotion of weight loss, individuals indicated self-reported weight loss from pre- to post-intervention that likely contributed to improvements in HRQL and symptom burden.

While the study program is inherently confounded due to its multi-faceted design, including social support, lifestyle education, medical supervision, and dietary guidance, the profound improvements observed in the quality of life and symptom burden seem to indicate a synergistic and compounding benefit from the inclusion of multiple therapeutic elements.

There are currently no published studies assessing the utilization of a comprehensive dietary and lifestyle intervention in participants with HT, making it difficult to provide an analysis of comparative or expected treatment effects. Winther et al. assessed the role of thyroxine treatment over a six-month period to improve quality of life in a population of 78 individuals newly diagnosed with HT and either subclinical hypothyroidism (4 µIU/mL < TSH < 10 µIU/mL), n = 66, or overt hypothyroidism (TSH > 10 µIU/mL ), n = 12 [3]. The authors noted that despite optimal medical management over the six-month period, only the SF-36 subscales of vitality, physical role functioning, and mental health showed statistically significant changes [3]. When examining the data from the 58 participants completing the six-month study, it should be noted that the increases in these three domains ranged from only 3%-8%, correlating to a minimal effect size as well as a potentially insignificant change in clinical status [3].

While we cannot compare SF-36 statistics from the AIP intervention directly with those from Winther et al., as we could not assume all SF 36 subscale data sets from the AIP intervention to be normally distributed and thus could not accurately calculate respective means and SDs, it is worth examining some of the more notable pre- to post-intervention changes for specific SF-36 subscales between the AIP intervention and the study group from Winther et al.

In examining the relative magnitude of changes for the SF-36 subscales: physical role functioning, vitality, mental health, and general health from the current intervention, the study authors observed a remarkable increase in physical role functioning scores from a median of 25, IQR 88, pre-intervention to a post-intervention median of 100, IQR 50, corresponding to a median difference of 50, IQR 75. Similar large-magnitude changes were noted when examining median values pre- and post-intervention for the vitality, mental health, and general health subscales. The pre-intervention median vitality subscale score was noted to be 23, IQR 19, however, post-intervention, the median vitality subscale score increased to 58, IQR 34, with a median difference of 33, IQR 29. The pre-intervention median mental health subscale score was noted to be 54, IQR 25, however, post-intervention, the median mental health subscale score increased to 78, IQR 19, with a median difference of 22, IQR 12. The pre-intervention median general health subscale score was noted to be 40, IQR 26, however, post-intervention, the median general health subscale score increased to 70, IQR 35, with a median difference of 28, IQR 21.

When comparing the magnitude of change noted for these three SF-36 subscales between the study from Winther et al. and the AIP intervention, it is important, first, to note the small and underpowered sample size of the AIP study. Additionally, the vitality subscale scores in the AIP trial at baseline were lower when compared to the baseline scores of participants in the Winther et al. trial, with both study populations becoming clinically equivalent post-intervention.

Mental health scores in this trial at baseline were clinically similar to those of Winther et al. (AIP pre-intervention median = 54, IQR 25; Winter et al. pre-intervention mean = 47 (SD 9), however, at post-intervention, there was a marked difference between these two study groups with the median mental health score post-intervention from the AIP study being equal to 78 (IQR 19) while the mean mental health score post-intervention from the Winther et al. trial was 50 (SD 10) [3]. When comparing the other SF-36 domains, greater improvements were also seen in the physical functioning, bodily pain, emotional role functioning, and social role functioning scales for participants in the AIP trial as compared to those in the Winther et al. trial [3].

In examining the bodily pain SF-36 subscale in the AIP trial, there was a notable increase from a pre-intervention median of 68 (IQR 22) to 78 (21) at post-intervention (p = 0.0112) as compared to Winther et al.: 52 (SD 12) pre-intervention to 55 (SD 10), p > 0.05 following six months of levothyroxine therapy [3]. In reviewing the specific subscales of the MSQ, it appeared that the improvements seen in bodily pain as measured by the SF-36 were primarily related to improvements in joint pain, muscle aches, and headaches as indicated more specifically by scores from the MSQ. Given the current concerns surrounding opioid misuse/overuse in those suffering from chronic pain conditions, including individuals with HT, multi-dimensional, non-pharmacologic interventions, such as the AIP dietary and lifestyle intervention utilized in this trial, may provide clinicians with novel, efficacious, and low-risk treatments for chronic pain.

Improvements in quality of life and symptom burden may be of critical benefit for both patients and clinicians, as it may help increase trust in providers as well as adherence to continued medical and lifestyle therapy. Despite prior work indicating that quality of life could be negatively impacted by restrictive diets, this study suggests that quality of life was not negatively impacted but markedly enhanced [10].

The reason for our conflicting findings regarding changes in quality of life, as previously suggested, may be due to the AIP study’s multi-dimensional treatment design involving frequent monitoring and interactions between participants with the team of health coaches and NTPs via a private Facebook group. Research on cancer survivors has shown preliminary evidence linking increased fruit and vegetable intake to increased social support as well as feelings of hope, possibly indicating a mechanism by which social support alone can improve one’s overall food choices [21].

Additional research indicates a strong association between a person’s quantity and quality of social interactions and their perceived health and quality of life [22]. It is unlikely, however, that given the profound improvements in the physical role functioning as well as vitality and general health subscales of the SF-36 that social interaction alone, whether between study participants themselves or between study participants and the multi-disciplinary team could account for all of the observed improvements in quality of life as well as overall symptom burden.

Limitations to the study include its small sample size, the lack of a control group, the lack of blinding, the possibility for selection bias of participants enrolling in the study, as well as response bias from participants regarding their weights. Additional limitations include the use of a medical symptoms questionnaire that has yet to be validated in large populations as well as the potentially transient nature of the participant’s symptoms being documented by the questionnaire. Further limitations to this study include the lack of data collection on physical activity, sleep, social support, stress management, or the effect that eliminated foods would have had if they were to be reintroduced systematically.

## Conclusions

Our pilot study suggests that an online, community-based AIP diet and lifestyle program facilitated by a multi-disciplinary team can significantly improve HRQL and symptom burden in middle-aged female subjects with HT. While there were no statistically significant changes noted in thyroid function or thyroid antibodies, the study’s findings suggest that AIP may decrease systemic inflammation and modulate the immune system, as evidenced by the decreases in average hs-CRP. Dietary and lifestyle changes may be a significant life stressor, but the use of health coaches and NTPs, in addition to nutritionally trained physicians, may offset this and provide an increase in quality of life. Larger randomized controlled trials are necessary to validate these findings and examine long-term follow-up, adherence, and any adverse events during the elimination and/or maintenance phase of AIP. Given the low-risk nature of the AIP dietary and lifestyle intervention as well as the improvements seen in HRQL and the participants’ symptom burden, further study in larger populations of individuals with HT implementing AIP as part of a multi-disciplinary diet and lifestyle program is warranted.

## Appendices

Case summaries and exploratory analyses

Case 1 

Case 1 involved a 29-year-old female with a past medical history of chronic rhinosinusitis. She was on multiple anti-inflammatory and allergy medications in addition to T4 and T3 therapy. She sought to adopt sustainable dietary, exercise, and stress management practices from participating in the study. She enrolled with a very high symptom burden (MSQ = 126) with the worst symptoms related to chronic rhinosinusitis, dermatological, musculoskeletal, and gastrointestinal systems. Her complaints specifically consisted of acne, dry skin, hair loss, joint and muscle aches, belching, bloating, and alterations in bowel habits, including constipation and diarrhea. She additionally complained of excessive weight, food cravings, compulsive eating, as well as cognitive and mood concerns with poor concentration, poor memory, anxiety, and depressed mood. Her initial FFQ revealed dietary patterns consisting of gluten-free refined products, refined potatoes, a variety of fruits, vegetables, processed and unprocessed meats, and dairy substitute products. She endorsed a predilection and craving for carbohydrate-rich foods.

Initial laboratory findings were skewed and invalid, as the participant was actively sick with an acute on chronic sinus infection. She was taking antibiotic medications at the time of the laboratory evaluation, however, there was some concern that the participant was possibly overmedicated with a suppressed TSH and elevated T4 and T3. The use of urinary organic acid testing revealed an increased need for vitamin B supplementation, most noticeably folate, as suggested by elevated formiminoglutamic acid (FIGLU) [23]. The participant also had a significantly elevated plasma copper. Stool testing revealed decreased short-chain fatty acids (SCFAs), most noticeably butyrate. SCFAs consist primarily of acetate, butyrate, and propionate, which are physiologically active byproducts produced via the fermentation of soluble dietary fibers and resistant starches by commensal bacteria throughout the colon [24]. Their concentrations vary along the length of the gastrointestinal tract with the highest levels in the cecum and proximal colon [24]. Butyrate acts as a fuel source for colonic enterocytes, with immune modulating properties through histone deacetylase inhibition, allowing for the suppression of macrophages and dendritic cells [24]. Additionally, SCFAs have an overall pH-lowering effect on the colon, allowing for the growth of beneficial bacteria, specifically Lactobacillus and Bifidobacterium [24].

At week six of the program, the participant was counseled to increase the consumption of folate-rich foods, such as organ meats and leafy greens, and experiment with eliminating foods high in fermentable oligosaccharides, disaccharides, monosaccharides, and polyols (FODMAPs) for gastrointestinal symptom relief [25]. She was encouraged to explore whether fermented foods exacerbated her allergy symptoms. She was instructed to consider lowering her thyroid medication dose given the concerning elevations found at study onset and to monitor for potential signs and symptoms of hyperthyroidism.

Following the program, the participant’s MSQ decreased from 126 to 43, with residual symptoms primarily related to her continued sinus and allergy complaints. She reported the exacerbation of allergy symptoms with fermented foods, a 12-pound weight loss, and the start of corticosteroid treatment just prior to final laboratory testing given another sinus infection. She later notified the medical team of further imaging revealing an anatomic abnormality of her maxillosinus structure and pursuit of corrective surgery.

A review of her second FFQ documenting the 10-week program showed strict adherence to the AIP diet with the elimination of refined carbohydrates, white potatoes, processed meats, eggs, nuts, grains, and dairy, as well as increased consumption of unprocessed meat, vegetables, and fruit, and the new inclusion of coconut, plantains, cassava flour, and maple syrup as the only sweetener. The participant’s exit survey revealed improvements in sleep, the beginning of an exercise program, and improvements in body composition. Laboratory testing revealed continued suppressed TSH with elevated total and free T4. The participant notified the team that she had previously decreased her thyroid medication, Synthroid, from 112 mcg to 100 mcg at week six followed by a further decrease to 88 mcg just prior to her final interview at week 10. She reported still experiencing signs concerning for hyperthyroidism and was planning to pursue an additional decrease in medication in conjunction with further treatment for chronic sinusitis. 

Review of repeat nutritional testing showed stable, but continued, elevation in her FIGLU as well as plasma copper. Repeat stool testing showed continued low SCFAs.

Case 2 

Case 2 involved a 41-year-old female with a history of depression, the use of selective serotonin reuptake inhibitor (SSRI) medication, and T4/T3 therapy. Her goals were to lose weight, improve food cravings, improve energy, and adopt a healthier lifestyle as a result of the study program. She entered the program with an MSQ of 98. Her worst symptoms affected the head, ears, eyes, nose, throat (HEENT), dermatological, musculoskeletal, neuropsychological, and genitourinary (GU) systems. More specifically, she complained of excessive mucus production in her pharynx, throat irritation, dry skin, hair loss, hyperhidrosis, joint aches and stiffness, anxiety, mood swings, irritability, excessive weight gain, food cravings, and compulsive and binge eating behaviors. Her initial FFQ revealed a diet consisting of refined grain products, refined potatoes, a variety of fruits, vegetables, regular processed and unprocessed meats, dairy, and eggs. She reported cravings for carbohydrate-rich foods and the regular consumption of sugar-sweetened soda beverages.

Initial laboratory findings revealed an elevated TSH at 4.75 μIU/mL and hs-CRP of 6.97 mg/L. Initial organic acid nutritional testing showed elevated suberic and adipic acids as well as an increased need for multiple B vitamins, most noticeably riboflavin as suggested by elevated glutaric acid [26]. Red blood cell (RBC) analysis for polyunsaturated fatty acid (PUFA) composition revealed elevations in omega-6 fatty acids and the low end of normal omega-3 fatty acids, resulting in a disturbed omega-3:omega-6 ratio. Stool testing revealed no pathogenic organisms, however, there was evidence of lipid malabsorption as measured by fecal phospholipids and cholesterol.

At week six of the program, the participant was counseled to increase the consumption of fish rich in omega-3 fatty acids, folate-rich foods, as well as glycine-rich foods such as bone broth. She was encouraged to explore the use of AIP-complaint digestive bitters to assist in the digestion and absorption of nutrients [27].

Following the program, the participant’s MSQ decreased from 98 to 12 with no further severe symptoms. She reported a 15-pound weight loss, improvements in energy, the cessation of soda consumption, and the beginning of a formal exercise regimen. 

A review of her second FFQ documenting the 10-week program showed strict adherence to the AIP diet with the elimination of refined carbohydrates, potatoes, eggs, legumes, coffee, nuts, seeds, grains, and dairy, as well as increases in the consumption of unprocessed meat, vegetables, fruit, avocado, and sweet potatoes.

Laboratory testing revealed a decrease in her TSH to 2.34 μIU/mL, with otherwise stable hormone levels and unchanged thyroid antibodies. Her hs-CRP decreased from 6.97 to 5.02 mg/L, however, it was noted to be a significant outlier (despite the decrease) when compared to the group average and was not included in the post-hoc secondary data analysis.

A review of repeat nutritional testing showed the improvement and normalization of previously elevated suberic and adipic acids and the presence of ketone bodies. The participant was noted with a deficiency in folate as suggested by a markedly elevated FIGLU [23]. The participant saw a decrease in overall omega-6 RBC volume and improved omega-3:omega-6 RBC ratio. Repeat stool testing showed resolved lipid malabsorption and normalized secretory IgA.

Case 3 

Case 3 involved a 34-year-old female with no significant past medical history. She began the study, taking only T3 replacement therapy at approximately 5 mcg four to six times daily. She sought to improve her dietary patterns, removing foods she was sensitive to, improve sleep, as well as begin a formal stress management program with yoga and meditation. She entered the program with a high MSQ score of 83, with the worst symptoms affecting the HEENT, dermatological, gastrointestinal, and neuropsychological systems. More specifically, she complained of dark circles under her eyes, sinus congestion, sneezing, acne, constipation, bloating, fatigue, lethargy, poor concentration, decreased memory, indecisiveness, depressed mood, excessive weight gain, food cravings, and behaviors of compulsive and binge eating. Her initial FFQ revealed a diet consisting of occasional gluten-free products and a variety of fruits, vegetables, potatoes, unprocessed meats, eggs, and infrequent dairy except for ice cream.

Initial laboratory findings revealed a TSH of 3.55 μIU/mL with low free T4 (fT4 = 0.31 ng/dL). Initial thyroid peroxidase (TPO) antibodies were 135 IU/mL and anti-thyroglobulin antibodies (TGA) were 2.0 IU/mL. Given these findings, the participant was counseled to begin either T4 only or combination T4/T3 therapy based on her weight. Initial organic acid nutritional testing showed an increased need for vitamin B6 as suggested by elevated xanthurenic acid [28]. RBC analysis for PUFA composition revealed elevations in omega-6 fatty acids, specifically linoleic acid and gamma-linolenic acid, and low normal omega-3 fatty acids, specifically DPA and DHA. This caused a disturbed omega-3:omega-6 ratio. Stool testing was with microscopic, and culture analysis revealed rare Blastocystis hominis and an overgrowth of Klebsiella oxytoca.

At week six of the program, the participant was counseled to continue increasing the consumption of omega-3 fatty fish, organ meats, and foods rich in vitamin B6. Additionally, she was encouraged to explore the inclusion of fermented foods.

Following the program, the participant’s MSQ decreased from 83 to 10 with no further severe symptoms. She reported an overall 10-pound weight loss with increased energy. She stated she was overall much happier with the resolution of brain fog as well as gastrointestinal complaints, including bloating, belching, and gas. She also reported improvements in her skin and acne.

A review of her second FFQ documenting the 10-week program revealed strict adherence to the AIP diet with the elimination of refined carbohydrates, potatoes, eggs, legumes, nuts, seeds, grains, and dairy, as well as increases in the consumption of unprocessed meat, vegetables, fruit, and avocado, and the new regular inclusion of coconut products, plantains, homemade liver pate, and bone broth.

Laboratory testing revealed an increase in TSH to 7.35 μIU/mL, however, during the final exit interview, the participant noted decreasing her use of T3 replacement to only 5 mcg one to two times daily, in addition to not starting any T4 replacement. An examination of her hormone levels showed an increase of free T4 from 0.31 to 0.77 ng/dL and total T4 from 2.0 to 5.0 μg/dL. T3 levels had maintained with the normal range. As the participant was not taking any replacement T4 and had decreased total T3 replacement use, the authors speculated that her thyroid began producing increased amounts of T4 due to decreased exogenous suppression and improvements in endogenous synthesis. The authors additionally speculate that the dietary intervention, in conjunction with decreased exogenous suppression, contributed to the participant’s increased T4 levels. As the participant decreased her use of thyroid replacement medications significantly during the intervention, her thyroid function laboratory data could not be included in the final data analysis. It should be noted, however, that the participant’s TGA normalized to <0.9 IU/mL and TPO antibodies decreased from 135 to 107 IU/mL.

A review of repeat nutritional testing revealed significant improvements in her RBC PUFA, analysis with increases in RBC eicosapentaenoic acid (EPA), docosapentaenoic acid (DPA), and docosahexaenoic acid (DHA) and decreases and normalization of RBC linoleic acid and gamma-linolenic acid. Repeat stool testing showed no overgrowth of pathogenic organisms with no noticeable lipid malabsorption. There was still overgrowth of additional Klebsiella species but no microscopic visualization of Blastocystis hominis.

At the end of the study during the exit interview, given her significant improvements, the participant informed the study team of her decision to continue her use of Liothyronine 5 mcg one to two times daily and repeat thyroid testing in three months.

Case 4 

Case 4 involved a 38-year-old female with no additional, significant past medical history who began the study taking 100 mcg of Synthroid. She wanted to improve her diet, sleep, weight, and energy and begin a formal exercise practice. She entered the program with a moderate symptom burden (MSQ = 55) with the worst symptoms affecting her dermatological, gastrointestinal, musculoskeletal, and neuropsychological systems. More specifically, she complained of acne, hair loss, dry skin, constipation, belching, bloating, joint pain, stiffness, muscle aches, fatigue, tiredness, lethargy, poor concentration, insomnia, increased weight, food cravings, compulsive eating, and binge eating. Her initial FFQ revealed a diet consisting of infrequent gluten-containing products, a variety of fruits, vegetables, regular unprocessed and processed meats, eggs, dairy, potatoes, one to three alcoholic beverages weekly, corn-based products, water, kefir beans, and legumes. Prior to enrollment, she had been taking multiple supplements containing various vitamins and minerals, a probiotic, occasional fish oil, evening primrose oil, and collagen peptides.

Initial laboratory findings revealed a TSH of 2.85 μIU/mL, TPO antibodies of 180 IU/mL, TGA of 603.5 IU/mL, and a slightly elevated hs-CRP at 2.64 mg/L. Initial organic acid nutritional testing showed balanced nutritional markers likely related to her previous and ongoing supplementation. RBC analysis for PUFA composition revealed a high normal omega-6 fatty acid profile, resulting in a disturbed omega-3:omega-6 ratio. Stool testing showed low normal SCFAs and an overgrowth of Klebsiella pneumonie and Candida albicans. There was no evidence of lipid malabsorption.

At week six of the program, the participant was counseled to increase consumption of omega-3 fatty fish and animal protein. She was encouraged to consider including fermented foods and removing foods high in FODMAP to improve her constellation of gastrointestinal symptoms, including belching, bloating, and irregular stools [25].

Following the program, the participant’s MSQ decreased from 55 to 28 with the only continued frequent symptom of hair loss. She reported a 15-pound weight loss with increased energy, improved sleep, decreased food cravings, improved compulsive eating or overeating, and improved cognitive functioning.

A review of her second FFQ documenting the 10-week program suggested strict adherence to the AIP diet with the elimination of refined carbohydrates, potatoes, eggs, legumes, nuts, seeds, grains, and dairy as well as increases in the consumption of unprocessed meat, vegetables, fruit, and the inclusion of coconut products, gelatin, collagen, sauerkraut, and kombucha.

Laboratory testing revealed a decrease in her TSH to 2.06 μIU/mL with an increase of free T4 from 1.4 to 1.59 ng/dL and total T4 from 8.3 to 9.8 μg/dL. T3 levels maintained within the normal range. Her hs-CRP was still elevated at 2.90 mg/L with TGA and TPO antibodies also remaining elevated and clinically unchanged.

A review of repeat nutritional testing revealed continued stability in vitamin and mineral markers with mild improvements in her RBC PUFA analysis, increased omega-3 RBC volume, and decreased omega-6 RBC volume. The participant also had elevated ketones related to weight loss and a low carbohydrate dietary pattern. Repeat stool testing revealed no growth of either K. pneumonie or C. albicans.

Case 5 

Case 5 involved a 26-year-old female with no significant past medical history, who enrolled in the study on 75 mcg of Synthroid. Her primary goal for participating in the program was to conceive a child. She had reported to the medical team prior to the study that she had been having significant difficulty in becoming pregnant. Her baseline MSQ was low (MSQ = 37) and was the lowest symptom score of any member of the study. Initial thyroid testing revealed a normal TSH and thyroid hormone levels with elevations in both TPO antibodies and TGA. Her hs-CRP was within normal limits at 0.65 mg/dL. Initial organic acid nutritional testing revealed no specific vitamin or mineral needs. Stool testing was notable for lipid malabsorption and low SCFAs.

The participant notified the study team during week eight that she had become pregnant. In conjunction with the study parameters and at the wishes of the participant, she discontinued participation in the study.

Case 6 

Case 6 involved a 44-year-old female with no significant past medical history and began the study on 88 mcg of Tirosint. She sought to “feel like herself again,” wanting to improve her mood and energy. She entered the program with a moderate MSQ (MSQ = 56), with the worst symptoms affecting the gastrointestinal, musculoskeletal, and neuropsychological systems. Specifically, these complaints consisted of constipation, belching, bloating, joint pain, stiffness, muscle aches, fatigue, tiredness, lethargy, poor concentration, comprehension, and memory, anxiety, irritability, mood swings, and headaches. Her initial FFQ revealed a diet consisting of gluten-free grains, a variety of fruits, vegetables, regular unprocessed meats, eggs, minimal dairy consumption, potatoes, vegetarian-based soy products, and the regular consumption of lentils, beans, and legumes. She endorsed taking multiple supplements containing various vitamins and minerals, fish oil, curcumin, and a flavonoid complex. 

Initial laboratory findings revealed normal thyroid function, however, there appeared to be low normal T3 levels and elevated reverse T3, indicating poor T4 to T3 conversion. Her hs-CRP was normal at 0.72 mg/L and TPO antibodies were elevated at 135 IU/mL. TGA was <0.9 IU/mL. Initial organic acid nutritional testing suggested deficiencies in riboflavin, as suggested by elevated glutaric acid, and vitamin B6, as suggested by elevated xanthurenate, respectively [26,28]. RBC analysis revealed a balanced PUFA composition with a high normal omega-3 RBC volume. The participant’s toxin profile revealed a markedly elevated whole blood mercury at 8.91 mcg/L. During the interview, the participant revealed consuming significant amounts of seafood and had several amalgam fillings with a history of recent dental work. Stool testing revealed low SCFAs and an overgrowth of Klebsiella oxytoca and Citrobacter freundii. There was no evidence of lipid malabsorption; however, there was no measurable fecal secretory IgA.

At week six of the program, the participant was counseled to consume animal protein and organ meats and increase her intake of vitamin B6-rich foods as well as fermented foods. Following the program, the participant’s MSQ decreased from 56 to 33 with an improvement in energy, headaches, joint pains, and cognition. She continued to report similar gastrointestinal symptoms of constipation, bloating, and belching in the setting of an overall excellent rating for her health.

A review of her second FFQ documenting the 10-week program showed strict adherence to the AIP diet with the elimination of refined carbohydrates, potatoes, eggs, legumes, nuts, seeds, grains, and dairy, as well as increases in the consumption of unprocessed meat, vegetables, and fruit, and the new regular inclusion of bone broth and collagen.

Laboratory testing revealed nearly identical values for thyroid function, thyroid antibodies, and hs-CRP, with no clinically significant changes. There was a decrease in white blood cell (WBC) count from 6.9 x10^3^/µL to 5.9 x 10^3^/µL as well as a decrease in neutrophil percentage from 69% to 59% and increase in lymphocyte percentage from 21% to 32%.

A review of repeat nutritional testing suggested a resolution of the riboflavin and vitamin B6 deficiency as suggested by normalized glutaric acid and xanthurenate [26,28]. Whole blood mercury decreased from an elevation above 8 mcg/L to within normal limits at 2.56 mcg/L. Repeat stool testing showed no growth of either K. oxytoca or C. freundii, an increase in total SCFAs, and a normal level of secretory IgA.

Case 7 

Case 7 involved a 33-year-old female with a past medical history of dyshidrotic eczema, elevated thyroid antibodies, including TPO and TGA. She was noted to be euthyroid without the utilization of thyroid hormone replacement medication prior to enrollment. She wanted to improve stress, improve symptoms in thyroid autoimmunity while continuing without the use of any thyroid replacement medication, improve eczema, and find a sustainable dietary template that met her needs. She entered the program with a very severe symptom burden (MSQ = 114) with the worst symptoms affecting the HEENT, dermatological, gastrointestinal, musculoskeletal, and neuropsychological systems. More specifically, she complained of watery eyes, circles under her eyes, hay fever, sinus problems, nasal congestion, frequent throat clearing, acne, dry skin, rashes, hair loss, constipation, belching, bloating, fatigue, tiredness, lethargy, poor concentration, confusion, poor memory, anxiety, excessive weight, food cravings, and compulsive binge eating. Her initial FFQ revealed a diet following a gluten-free and dairy-free template with a variety of fruits, vegetables, unprocessed and processed meats, eggs, and limitations on starches and potatoes. She was also taking numerous supplements prior to the study but had recently stabilized on magnesium and fish oil.

Initial laboratory findings revealed a TSH of 1.06 μIU/mL, TPO antibodies of 273 IU/mL, TGA of 4.8 IU/mL, and a normal hs-CRP at 0.43 mg/L. Initial organic acid nutritional testing showed numerous imbalances, including elevated adipic and suberic acids. The participant had significantly elevated levels of the ketone body beta-hydroxybutyrate (BHB) likely related to her low carbohydrate consumption. Riboflavin was deficient, as suggested by elevated glutaric acid [26]. She was otherwise balanced in her additional vitamin and mineral markers. The participant’s toxic element screen revealed elevated blood mercury at 4.74 mcg/L. Stool testing revealed additional imbalances, including marked lipid malabsorption, low SCFAs, and an overgrowth of Klebsiella pneumonie and Citrobacter freundii. Microscopic evaluation also revealed evidence of trophozoites of Dientamoeba fragilis. Fecal calprotectin and fecal secretory IgA were within normal limits.

At week six of the program, the participant was counseled to continue with increased consumption of omega-3 fatty fish, animal protein, and organ meats. She was counseled to take AIP-compliant digestive bitters to support improved lipid and nutrient absorption [27]. She was counseled on the removal of high FODMAP-containing foods given her gastrointestinal symptoms, including belching, bloating, and constipation [25].

Following the program, the participant’s MSQ decreased from 114 to 56 with continued eczema and rashes, despite improvement in acne. Constipation remained despite improvements in bloating and belching. She reported increased energy, decreased lethargy and apathy, minimal compulsive eating or overeating and improved cognitive functioning. In continuing discussions with the participant and at the suggestion of the study doctor, she reported secondary evaluations for constipation revealing dyssynergic constipation and pelvic floor muscular weakness.

A review of her second FFQ documenting the 10-week program showed strict adherence to the AIP diet with the only challenges occurring early in the first few weeks during a 10-day vacation. Laboratory testing revealed stability in her TSH at 1.13 μIU/mL with an increase of free T4 from 1.11 to 1.19 ng/dL and free T3 from 2.2 to 2.5 pg/mL. Her hs-CRP remained low at 0.38 mg/L, TGA relatively unchanged at 4.8 IU/mL, and there was a significant decrease in TPO antibodies from 273 to 190 IU/mL.

A review of repeat nutritional testing revealed continued stability in vitamin and mineral markers with the normalization of elevated glutaric acid [26], as well as the normalization of previously elevated suberic and adipic acids. The participant no longer had elevated ketone bodies, likely related to the inclusion of additional carbohydrates and certain starches during the program. The previously elevated whole blood mercury had fallen from 4.74 to 1.79 mcg/L.

Repeat stool testing showed the resolution of previously noted severe lipid malabsorption. There was no microscopic evidence of D. fragilis. She was noted to still have low total SCFAs and overgrowth of previously noted Citrobacter species. Fecal calprotectin and fecal secretory IgA remained normal. Upon completion of the study, the participant continued without the utilization of thyroid hormone replacement.

Case 8 

Case 8 involved a 33-year-old female with a past medical history of attention deficit hyperactivity disorder (ADHD) and depression who was recently diagnosed with subclinical hypothyroidism and autoimmune thyroiditis via elevated TPO antibodies and TGA. She began the study program without the utilization of thyroid replacement medication. She wanted to improve stress, delay or stop the progression of any thyroid autoimmunity, continue without the use of any thyroid replacement medication, address poor sleep and low energy, and improve diet. She entered the program with a very severe symptom burden (MSQ = 108) with the worst symptoms affecting dermatological, gastrointestinal, musculoskeletal, and neuropsychological systems. More specifically, she complained of dry skin, flushing, hyperhidrosis, nausea, abdominal pain, bloating, joint pain, muscle aches, stiffness, fatigue, tiredness, lethargy, poor concentration, confusion, poor memory, mood swings, irritability, anxiety, excessive weight gain, food cravings, compulsive and binge eating. Her initial FFQ revealed the intake of chicken, beef, and, occasionally, fish, restrictions on fruit intake - only berries, a variety of vegetables, grains, potatoes, and some candy and chocolate. She used a daily B complex as well as magnesium in addition to the prescribed medications Vyvanse, Fluvox and low dose naltrexone (LDN).

Initial laboratory findings revealed a TSH of 13.45 μIU/mL, TPO antibodies of 185 IU/mL, and TGA of 1.8 IU/mL. Her hs-CRP was elevated at 2.59 mg/L and all total and free thyroid hormone levels were within normal limits. Initial organic acid nutritional testing showed a likely folate deficiency, as suggested by a markedly elevated FIGLU [25-26]. Plasma copper was high normal at 151.5 mcg/dL. RBC volume of omega-3 fatty acids, including EPA, DPA, and DHA, were within normal limits. Her toxic element screen revealed elevated whole blood mercury at 4.82 mcg/L, and stool testing revealed an overgrowth of Klebsiella pneumonie. Fecal calprotectin and fecal secretory IgA were within normal limits.

At week six of the program, the participant was counseled to continue with the previous consumption of omega-3 fatty fish and increase the intake of various AIP-compliant foods, including animal protein and organ meats. Additionally, she was encouraged to explore the removal of high FODMAP-containing foods given her gastrointestinal symptoms, including abdominal pain and bloating [25].

Following the program, the participant’s MSQ decreased slightly from 114 to 85, with continued symptoms in the gastrointestinal, dermatological and musculoskeletal systems despite improvements in acne, bloating, belching, energy, joint pain, compulsive eating or overeating, and cognitive functioning.

A complete objective analysis of the participant’s health following the program could not be conducted, however, as she was lost to follow-up, unable to complete her final FFQ, stool, and organic acid testing. Review of food journals during the study revealed challenges with frequent travel to various social engagements and consumption of foods outside of the AIP template.

Final laboratory testing was also difficult to interpret, as the participant was acutely ill with an infection as evidenced by an elevated WBC count, platelets and hs-CRP. In terms of thyroid hormone fluctuations, the participant’s TSH had risen to 31.92 μIU/mL with slight increases in free and total hormones (T3 and T4). TGA had increased to 4.5 IU/mL, but TPO antibodies had decreased from 185 to 123 IU/mL. As of the final communication with the participant at the end of the study program, she has not started thyroid replacement medication.

Case 9 

Case 9 involved a 36-year-old female with a past medical history of ruptured ovarian cysts, dysmenorrhea concerning for endometriosis who enrolled in the study on 45 mg of NP thyroid. She wanted to improve stress, joint pain, fatigue, bloating, IBS-like symptoms, minimize symptom exacerbation during menstrual cycles, and decrease hair loss. She entered the program with a very severe symptom burden (MSQ = 119) with the worst symptoms affecting the HEENT, dermatological, gastrointestinal, genitourinary, musculoskeletal, and neuropsychological systems. Specifically, symptoms included headaches, pruritic eyes, dark circles under her eyes, pruritic ears, otalgia, hay fever, sinus problems, excessive mucus, hair loss, constipation, belching, bloating, joint pain, stiffness, muscle aches, fatigue, tiredness, poor concentration, coordination and memory, anxiety, mood swings, irritability, frequent urination, and discharge. Her initial FFQ revealed a diet low in total animal products with the majority of animal protein coming from eggs and chicken. She consumed numerous fruits and vegetables, no dairy, minimal gluten consumption with the majority of grain-based foods being gluten-free or rice-based. She took magnesium and vitamin D as supplements and used histamine-2-receptor antagonists as needed for worsening allergic symptoms.

Initial laboratory findings revealed a TSH of 2.79 μIU/mL, TPO antibodies of 471 IU/mL, TGA of 3.4 IU/mL, and hs-CRP within normal limits at 0.76 mg/L. Initial organic acid nutritional testing revealed elevated adipic acid. RBC analysis of omega-3s was within normal limits due to the regular consumption of fatty fish. Plasma copper was low at 70.6 mcg/dL and RBC magnesium was also low despite supplementation at 27.8 mcg/g. Vitamin D was noted to be 83.4 ng/dL and the participant was instructed to discontinue the supplementation of 10,000 IU daily until re-testing in 12 weeks. Her toxic element screen revealed elevated whole blood mercury at 4.66 mcg/dL. Stool testing revealed low SCFAs and an overgrowth of Citrobacter species. Fecal calprotectin was normal and fecal secretory IgA was undetectable.

At week six of the program, the participant was counseled to continue with increased consumption of omega-3 fatty fish and animal protein, including organ meats. She was guided on foods highest in copper and magnesium. Additionally, she was encouraged to explore the removal of high FODMAP-containing foods, given her collection of gastrointestinal symptoms, including bloating, abdominal pain, and constipation [25]. She was additionally given education on monitoring allergic symptoms around the intake of fermented foods.

Following the program, the participant’s MSQ decreased dramatically from 119 to 32, with continued symptoms of painful menstruation and other fluctuations during ovulation. The participant reported complete elimination of joint pain, as well as anxiety, depressed mood, and impaired cognitive functioning. Multiple allergic symptoms, including sinus complaints and hay fever, resolved without the use of medication. She reported increased energy, decreased lethargy and tiredness, as well as resolved frequent urination and insomnia.

A review of her second FFQ documenting the 10-week program showed strict adherence to the AIP diet with regular consumption of bone broth, organ meats, coconut, and AIP-allowed starches. Laboratory testing revealed stability in her TSH at 3.06 μIU/mL with all total and free hormone remaining in the low normal ranges. Her hs-CRP remained low and decreased to 0.36 mg/L. TGA remained unchanged at 3.6 IU/mL as did TPO antibodies measured at 481 IU/mL. 

Review of repeat nutritional testing showed improvement in multiple vitamin and mineral markers with normal levels of all B vitamins. Previously elevated adipic acid had normalized. Plasma copper had increased into the normal range, however, RBC magnesium still remained unchanged. The previously elevated whole blood mercury had fallen slightly from 4.74 to inside the normal range at 4.19 mcg/L.

Repeat stool testing showed the normalization of SCFAs. The previous overgrowth of Citrobacter species was resolved. There was now a detectable and normal level of fecal secretory IgA.

The participant continued following the dietary pattern and increased her thyroid replacement medication to 60 mg of NP thyroid while seeking further evaluation and support for ongoing menstrual complaints.

Case 10

Case 10 involved a 39-year-old female with a past medical history of mild transaminitis who began the program on 75 mcg of Levothyroxine and 5 mcg of Liothyronine. She wanted to improve stress, energy, bloating, IBS-like symptoms, sleep, and hair loss, and minimize/lower inflammation and lose weight. She entered the program with a moderate to severe symptom burden (MSQ = 89), with the worst symptoms affecting the HEENT, dermatological, gastrointestinal, musculoskeletal, and neuropsychological systems. Specific complaints consisted of headaches, dizziness, pruritic eyes, tinnitus, otalgia, excessive clearing of throat, sore throat, acne, dry skin, hair loss, diarrhea, constipation, belching, bloating, heartburn, abdominal pain, joint pain, stiffness, muscle aches, fatigue, tiredness, lethargy, anxiety, mood swings, irritability, and depressed mood. Her initial FFQ revealed the majority of animal protein coming from chicken and fish, numerous fruits and vegetables, minimal to no dairy, nut-based dairy substitutes, a mixture of whole grain and processed grain foods, rice, coffee, and coconut products. She took numerous vitamin and mineral supplements, including selenium, zinc, magnesium, biotin, iron with vitamin C, B12, and vitamin D.

Initial laboratory findings revealed a TSH of 2.09 μIU/mL, TPO antibodies above the reference range at >600 IU/mL, and TGA at 2.1 IU/mL. Her hs-CRP was elevated at 2.42 mg/L. Initial organic acid nutritional testing revealed elevated suberic acid. She has elevated FIGLU suggestive of folate deficiency [23] as well as elevated xanthurenate, suggesting B6 deficiency [28]. RBC analysis of omega-3s was within normal limits and related to the participant’s regular consumption of fatty fish. Her toxic element screen revealed elevated whole blood mercury at 7.58 mcg/L and elevated selenium at 427 mcg/L. Upon further questioning, the elevated selenium may have been caused by 400 mcg of selenium supplementation every day for over a year. She was asked to discontinue supplementation given the concern for toxicity. Her initial blood chemistry revealed an elevated ALT at 57 IU/L with normal AST at 32 IU/L. Initial stool testing revealed low SCFAs and an overgrowth of Citrobacter freundii and fungal Geotrichum species. Fecal calprotectin was normal.

At week six of the program, the participant was counseled to continue with increased consumption of omega-3 fatty fish as well as animal protein, focusing on red meat as well as organ meats. She was educated on foods highest in copper and magnesium and encouraged to decrease high FODMAP-containing foods given her gastrointestinal symptoms of bloating, abdominal pain, diarrhea, and constipation [25]. She was instructed on folate and vitamin B6-rich foods, including organ meats, beef, leafy greens, spinach, mushrooms, and beets, as well as the use of AIP-compliant bitters to improve digestion [27].

Following the program, the participant’s MSQ decreased dramatically from 89 to 6, with no frequent or severe symptoms. The participant reported complete elimination of joint pain, anxiety, depressed mood, irritability, and gastrointestinal symptoms. She reported markedly improved sleep, energy, skin, lethargy, feelings of soreness, and a 14-pound weight loss.

A review of her second FFQ documenting the 10-week program revealed strict adherence to the AIP diet with regular consumption of bone broth, organ meats, coconut, and AIP starches. She reported multiple daily servings of cruciferous and leafy green vegetables and increased intake of animal protein.

Laboratory testing revealed a suppressed TSH at 0.535 μIU/mL and the participant reported concern for overmedication. Her hs-CRP decreased from 2.42 to 0.84 mg/L, TGA decreased slightly from 2.1 to 1.7 IU/mL, TPO antibodies remained above the lab reference range at >600 IU/mL, ALT normalized to 13 IU/L and AST decreased within the normal range to 16 IU/L.

Repeat nutritional testing revealed marked elevation in ketone bodies likely related to participant’s weight loss and dietary changes. FIGLU remained elevated, however, xanthurenate had normalized suggesting a resolved B6 deficiency [28]. Blood selenium remained elevated and previously elevated blood mercury fell from 7.58 to 6.29 mcg/L. Repeat stool testing revealed resolution of prior Geotrichum yeast overgrowth and continued overgrowth of C. freundii. The participant continued following the study following the dietary pattern and chose to decrease her thyroid replacement medication to 50 mcg of levothyroxine.

Case 11

Case 11 involved a 39-year-old female with no significant past medical, who began the program on 75 mcg of Levothyroxine. She wanted to decrease stress, improve energy, decrease pain and irritability, and lose weight. She entered the program with the highest symptom burden of any participant (MSQ = 132), with the worst symptoms affecting the HEENT, dermatological, cardiac, pulmonary, gastrointestinal, musculoskeletal, and neuropsychological systems. Specific complaints consisted of headaches, faintness, dizziness, watery eyes, blurred/tunnel vision, dark circles under her eyes, pruritic ears, ear drainage, stuffy nose, sinus problems, excessive mucus, sore throat, dry skin, hair loss, flushing, irregular heartbeat, chest congestion, diarrhea, bloating, abdominal pain, joint pain, stiffness, muscle aches, fatigue, tiredness, lethargy, restlessness, anxiety, poor concentration, poor memory, mood swings, irritability, depressed mood, and frequent urination. Her initial FFQ revealed the majority of protein coming from chicken and eggs, with infrequent fish, red meat, and pork. She consumed numerous fruits and vegetables, regular dairy consumption, minimal refined grains, with regular whole grain and potato consumption. She took a daily probiotic and multivitamin.

Initial laboratory findings revealed a high normal TSH of 4.08 μIU/mL. Initial TPO antibodies and TGA were both within normal ranges although the participant’s previous bloodwork from less than six months prior to the study showed elevated TPO antibodies. Her hs-CRP was normal at 0.98 mg/L. Initial organic acid nutritional testing was markedly abnormal with only vitamin C in the normal range. There were marked elevations in suberic and adipic acids. She had an elevated methylmalonic suggestive of vitamin B12 deficiency [29]. Glutaric acid was elevated, suggesting a riboflavin deficiency [26]. Xanthurenate was elevated, suggesting a B6 deficiency [28]. RBC analysis of omega-3 volumes was low, with elevations in omega-6 RBC volume resulting in a markedly disturbed omega-3:omega-6 ratio. Urinary amino acids suggested significant protein catabolism given elevations in multiple essential and non-essential amino acids. Her toxic element screen revealed an elevated whole blood lead at 3.41 mcg/dL. Stool testing revealed low SCFAs and an overgrowth of Morganella morganii. While fecal calprotectin was normal, there was no identifiable fecal secretory IgA.

At week six of the program, the participant was counseled to continue with consumption of omega-3 fatty fish as well as increase animal protein, focusing on red meat as well as organ meats. She was encouraged to try removing high FODMAP-containing foods, given her gastrointestinal symptoms of bloating and abdominal pain [25]. She was educated on riboflavin, vitamin B6, and vitamin B12-rich foods, and the use of AIP-compliant digestive bitters to aid digestion [27].

Following the program, the participant’s MSQ decreased dramatically from 132 to 8, with no frequent or severe symptoms. The participant reported complete elimination of joint pain and muscle aches, as well as anxiety, depressed mood, and irritability. Gastrointestinal symptoms completely resolved, she reported the disappearance of rash/hives, improved energy, decreased hunger, panic attacks, termination of headaches, and a six-pound weight loss, with minimal to no continued hair loss.

A review of her second FFQ documenting the 10-week program revealed strict adherence to the AIP diet with regular consumption of bone broth, organ meats, coconut, and AIP starches. She reported multiple daily servings of cruciferous and leafy green vegetables and increased intake of animal protein.

Laboratory testing revealed that her TSH had decreased from 4.08 to 2.15 μIU/mL, with an increase in free T4 from 1.19 to 1.47 ng/dL and a decrease in hs-CRP from 0.98 to 0.9 mg/L. TGA and TPO antibodies remained within the lab reference range. Review of repeat nutritional testing revealed a marked improvement in multiple domains with the only continued nutrient deficiency being B12 and riboflavin, as suggested by elevated methylmalonic acid [29] and glutaric acid [26]. Both methylmalonic acid and glutaric acid, however, had come down dramatically from severe elevations at pre-intervention to just outside the reference range at post-intervention. Previously elevated whole blood lead fell to within normal limits at 0.31 mcg/dL. Urinary amino acids had normalized, suggesting a resolution of the previously suspected catabolic physiology. Suberic and adipic acids were now in the normal range. There was a mild elevation in ketone bodies likely related to the participant’s dietary pattern and weight loss. RBC omega-3 volume remained low and imbalanced compared to omega-6 fatty acid volume.

Repeat stool testing revealed the resolution of previous M. morganii overgrowth with continued insufficiency of beneficial organisms and low SCFAs. There was an isolated elevated fecal phospholipid without other evidence of lipid malabsorption. Fecal calprotectin remained normal and fecal secretory IgA increased to within normal limits.

Case 12

Case 12 involved a 32-year-old female with a past medical history of iron deficiency and eczema who began the program on 125 mcg of levothyroxine. She wanted to decrease stress, improve fatigue and eczema, and address bloating and IBS-like symptoms. She entered the program with a moderately elevated symptom burden (MSQ = 83), with the worst symptoms affecting the HEENT, dermatological, gastrointestinal, genitourinary, musculoskeletal, and neuropsychological systems. Her complaints specifically consisted of headaches, dizziness, dark circles under her eyes, acne, dry skin, hair loss, belching, bloating, joint pain, stiffness, muscle aches, fatigue, tiredness, apathy, poor memory, indecisiveness, anxiety, mood swings, irritability, and depressed mood, as well as frequent/urgent urination. Her initial FFQ revealed a diet low in total animal products, numerous fruits and vegetables, occasional dairy, regular corn, and refined and whole gluten-containing grain products. Her supplements consisted of collagen and iron.

Initial laboratory findings revealed a TSH of 0.73 μIU/mL, TPO antibodies at 438 IU/mL, a TGA of 3.2 IU/mL, and a hs-CRP of 0.29 mg/L. Initial organic acid testing revealed elevated adipic and suberic acids and increased needs for riboflavin [26], vitamin B6 [28], folate [23], and vitamin B12 [29]. RBC analysis of omega-3s was within normal limits and related to the participant’s regular consumption of fatty fish. Urinary amino acids were elevated and suggested catabolic physiology given the participant’s vigorous, regular resistance and cardiometabolic exercise without sufficient rest. Her toxic element screen revealed elevated whole blood mercury at 4.50 mcg/L and lipid peroxides were also elevated, with low serum CoQ10.

Stool testing revealed low SCFAs and pancreatic insufficiency as measured by low fecal elastase [30]. There was also microscopic evidence of Blastocystis hominis, normal fecal calprotectin, and undetectable fecal secretory IgA.

At week six of the program, the participant was counseled to continue the consumption of animal protein, including red meat and organ meats as well as other foods high in thiamine, riboflavin, B6, folate, and B12. Additionally, she was encouraged to explore the inclusion of fermented foods and AIP-compliant bitters to support digestion [27].

Following the program, the participant’s MSQ decreased from 83 to 25, with symptoms of mood swings and her depressed mood improved. On the exit questionnaire, the participant reported complete resolution of eczema and joint complaints, improved energy, and satisfaction with the elimination of grains, including gluten-containing products as well as corn. The participant reported increased resilience amidst continued life stressors. She did not report changes in weight.

A review of her second FFQ documenting the 10-week program revealed strict adherence to the AIP diet, with significantly increased and regular consumption of unprocessed animal protein, coconut, and AIP starches.

Laboratory testing revealed suppression of her TSH to 0.23 μIU/mL. Her hs-CRP remained low at 0.32 mg/L and both TGA and TPO antibodies remained unchanged at 4.8 IU/mL and 452 IU/mL, respectively. Given the stability in the participant’s weight, but decreasing TSH to now suppressed levels, the authors speculated that the improvements in thyroid function were directly related to elements of the dietary and lifestyle intervention positively impacting thyroid hormone production and absorption of the participant’s replacement medication.

Review of repeat nutritional testing revealed normalization of suberic and adipic acids, FIGLU, and glutaric acid as well as xanthurenate. The participant persisted with borderline elevated methylmalonic acid, normalization of urinary amino acids, suggesting a reversal of previous catabolic physiology, and normalization of lipid peroxides. Previously elevated whole blood mercury fell into the normal range from 4.74 to 3.45 mcg/L.

Repeat stool testing revealed a continuation of low fecal elastase, as well as continued microscopic evidence of B. hominis. There was, however, a normalization of fecal secretory IgA, and fecal calprotectin remained within normal limits.

At the conclusion of the study, the participant decreased her thyroid replacement medication to 100 mcg of levothyroxine while seeking evaluation and support for pancreatic insufficiency.

Case 13

Case 13 involved a 44-year-old female who began the program on 125 mcg of Tirosint and 15 mcg of Liothyronine. She sought to reduce stress, improve fatigue and eczema, and address bloating and IBS-like symptoms. She entered the program with a moderately severe symptom burden (MSQ = 77) with the worst symptoms affecting the HEENT, dermatological, gastrointestinal, musculoskeletal, and neuropsychological systems. Specifically, her complaints consisted of headaches, dark circles under her eyes, dry skin, hair loss, diarrhea, constipation, belching, bloating, joint pain, arthritis, stiffness, muscle aches, fatigue, tiredness, apathy, poor memory, poor concentration, anxiety, mood swings, and irritability. Her initial FFQ revealed a diet high in animal products, including beef and pork, low intake of eggs and fatty fish, no gluten-based grains or dairy consumption, infrequent gluten-free grains, and numerous fruits and vegetables. Her supplements consisted of a probiotic, “adrenal adaptogens,” magnesium, glutamine, vitamin D, vitamin K2, dehydroepiandrosterone (DHEA), 5-HTP, and liposomal glutathione.

Initial laboratory findings revealed a TSH of 0.65 μIU/mL, TPO antibodies of 30 IU/mL, elevated TGA at 826.8 IU/mL, and a hs-CRP of 1.95 mg/L. Initial organic acid nutritional testing revealed mildly elevated suberic acid. There was suspicion for a folate deficiency, as suggested by elevated FIGLU [23]. Lipid peroxides were also elevated. Stool testing revealed no lipid malabsorption or pathogenic overgrowth. There were low normal SCFAs and a normal fecal calprotectin.

At week six of the program, the participant was counseled to continue consumption of fatty fish and organ meats. She was provided with guidance on folate-rich foods and to consider the inclusion of fermented foods.

Following the program, the participant’s MSQ decreased from 77 to 25, with notable improvements in joint pain, cognition, and mood. On the exit questionnaire, the participant reported continued challenges with stress without markedly noticeable changes in her overall health. The participant reported a desire to continue prioritizing stress management practices.

A review of her second FFQ documenting the 10-week program showed strict adherence to the AIP diet, with increased intake of certain fruits and vegetables and the use of AIP-compliant starches. Repeat laboratory testing revealed an elevated TSH of 5.07 μIU/mL while free and total hormones remained within the normal range. On questioning, the participant was surprised by the increased TSH, given some mild improvements in symptoms and no symptoms of worsening hypothyroidism. She reported that she had large fluctuations with her TSH in the past, with difficulty titrating medication and maintaining a stable TSH. Her hs-CRP decreased from 1.95 to 1.63 mg/L, TPO antibodies remained unchanged at 29 IU/mL and TGA rose slightly to 884.3 IU/mL.

Repeat nutritional testing revealed normalized adipic acid but slightly elevated suberic acid. FIGLU remained elevated, however, lipid peroxides normalized. Repeat stool testing revealed no changes outside of a notable overgrowth of K. pneumonie. Given the previous concerns for arthritis and the noticeable presence of K. pneumonie, the participant was counseled on testing for HLA-B27.

Case 14 

Case 14 involved a 33-year-old female, with no additional significant past medical history, who began the study taking 120 mg of NP thyroid. She sought to improve energy, decrease inflammation, and lose weight. Initial MSQ was 55, with the worst symptoms affecting the HEENT, dermatological, gastrointestinal, and neuropsychological systems. Complaints consisted of pruritic ears, watery/pruritic eyes, stuffy nose, sinus problems, hay fever, excessive mucus, hyperhidrosis, mild hair loss, diarrhea, constipation, bloating, fatigue, tiredness, infrequent poor concentration, comprehension and memory, and anxiety. Her initial FFQ revealed a diet consisting of a variety of fruits, vegetables, regular unprocessed and occasional processed meats, infrequent dairy with regular use of non-dairy creamer, both refined and whole grain products, rice, daily coffee, and weekly alcohol use. She endorsed taking between 5,000 to 10,000 IU vitamin D daily as well as 100 mcg of vitamin K2.

Initial laboratory findings revealed borderline low TSH at 0.42 μIU/mL and free and total T4 and T3 in the low normal range. Her hs-CRP was slightly elevated at 1.71 mg/L, TPO antibodies were slightly elevated at 99 IU/mL with TGA <0.9 IU/mL. Vitamin D was noted to be high normal at 81.8 ng/mL and serum calcium just outside the normal range at 10.3 mg/dL. Given the concern for hypercalcemia and hypervitaminosis D, the participant was asked to discontinue the use of vitamin D until reassessment at the end of the 10-week study.

Initial organic acid nutritional testing revealed an elevation in adipic acid. The participant had significantly elevated ketone bodies despite not following a low carbohydrate diet, which was concerning for possible cellular insulin resistance. Stool testing revealed very low SCFAs and an overgrowth of Klebsiella oxytoca, Pseudomonas aeruginosa, and Enterobacter cloacae. There was evidence of significant lipid malabsorption, as evidenced by elevated fecal phospholipids and fecal cholesterol. Fecal secretory IgA and calprotectin were within normal limits. 

At week six of the program, the participant was counseled to continue consuming animal protein, including organ meats. She was encouraged to explore the inclusion of fermented foods and the use of AIP-compliant digestive bitters to support digestion [27]

Following the program, the participant’s MSQ decreased from 56 to 12 with improvements in sleep, sustained energy, and reduction in HEENT symptoms, bloating, and only occasional loose stools. She reported a 12-pound weight loss and a desire to continue with stress management practices to support her health.

A review of her second FFQ documenting the 10-week program revealed strict adherence to the AIP diet, with the elimination of refined carbohydrates, potatoes, eggs, legumes, nuts, seeds, grains, and dairy, as well as the increased consumption of unprocessed meat, vegetables, and fruit and the new, regular inclusion of bone broth, sweet potatoes, fermented foods, and coconut-based products.

Repeat laboratory testing revealed a significantly suppressed TSH at 0.069 μIU/mL, a free T3 increase from 2.3 to 2.9 ng/dL, a decrease of TPO antibodies from 99 to 75 IUI/mL, TGA <0.9 IU/mL, a decrease in hs-CRP from 1.71 to 0.70 mg/L, and a normalization of vitamin D at 50.8 ng/mL.

Repeat stool testing revealed improved, but continued, concern for lipid malabsorption and low SCFAs. There was no growth of any of the previously identified potentially pathogenic organisms. On the exit interview, the participant was instructed to seek digestive enzyme supplement therapy and further work-up for continued lipid malabsorption.

Case 15

Case 15 involved a 43-year-old female, with no additional significant past medical history, who began the study taking 150 mg of Armour. She expressed a desire to improve her diet, improve sleep, lose weight, improve energy and cognition, and begin a structured exercise protocol. She entered the program with a severe symptom burden (MSQ = 103) with the worst symptoms affecting the HEENT, dermatological, gastrointestinal, genitourinary, musculoskeletal, and neuropsychological systems. More specifically, she complained of headaches, dizziness, dark circles under her eyes, pruritic ears and otalgia, acne, dry skin, rashes, hair loss, flushing, constipation, belching, bloating, abdominal pain, joint pain, stiffness, muscle aches, fatigue, tiredness, lethargy, poor concentration, poor coordination, indecisiveness, anxiety, mood swings, irritability, frequent urination, and insomnia. She additionally complained of excessive weight gain, food cravings, and water retention.

Her initial FFQ revealed a diet consisting of a variety of fruits, vegetables, regular unprocessed and processed meats, eggs, infrequent seafood, regular dairy consumption, potatoes, limited refined or whole grains or grain-based products, daily coffee, and weekly alcohol. The participant was not taking any supplements or additional medications.

Initial laboratory findings revealed a markedly suppressed TSH of 0.026 μIU/mL, with total and free hormone levels within normal ranges. TPO antibodies were noted at 141 IU/mL, TGA at 21.8 IU/mL, and hs-CRP was elevated at 1.85 mg/L. The participant was asked given the significantly suppressed TSH, to lower her medication dose to 120 mg.

Initial organic acid nutritional testing revealed borderline elevated methylmalonic acid, suggesting borderline vitamin B12 deficiency [29], as well as elevated suberic and adipic acids. Her toxic element screen revealed a slightly elevated whole blood tin at 0.45 mcg/L. Stool testing showed normal SCFAs, but an overgrowth of C. freundii and E. cloacae.

At week six of the program, the participant was counseled to continue with increased consumption of omega-3 fatty fish and animal protein, including organ meats, and explore the inclusion of fermented foods as tolerated.

Following the program, the participant’s MSQ significantly decreased from 103 to 36 with the only continued symptom of hair loss. She reported a 10-pound weight loss, increased energy, improved sleep, decreased food cravings, improved resilience, decreased bloating, and improved cognitive functioning.

A review of her second FFQ documenting the 10-week program revealed strict adherence to the AIP diet with the elimination of refined carbohydrates, potatoes, eggs, legumes, nuts, seeds, grains, and dairy, as well as increases in the consumption of unprocessed meat, vegetables, fruit, and regular inclusion of coconut products and AIP-approved starches. The participant did report some accidental consumption of gluten in beverages and processed products in the first few weeks of the program with noticeable negative effects on energy and stools that improved after the discovery and elimination of the gluten-containing products.

Repeat laboratory testing revealed TSH of 0.244 μIU/mL, with free and total hormone levels staying within normal limits, hs-CRP decreased to 0.94 mg/L, TPO antibodies decreased from 141 to 111 IU/mL, and TGA remained clinically unchanged.

Repeat nutritional testing revealed continued slight elevation in adipic acid. Methylmalonic acid remained elevated, suggesting B12 deficiency [29], however, previously elevated whole blood tin had normalized. The participant also now had slightly elevated ketones likely related to weight loss and lower carbohydrate diet.

Repeat stool testing revealed no evidence of lipid malabsorption and no overgrowth of previously identified potentially pathogenic organisms, however, there was a reduction in the predominant SCFA butyrate [23]. Upon the completion of the study, the participant decreased her medication from 120 mg Armour to 90 mg. Given the persistently elevated methylmalonic acid in the setting of normal and even increased animal protein intake, the participant was instructed by the study doctor to seek further diagnostic evaluation for potential autoimmune gastritis compromising B12 absorption.

Case 16

Case 16 involved a 26-year-old female, with no additional significant past medical history, who began the study without the use of thyroid replacement medication. She reported the use of the dietary supplement Standard Process Thyrotrophin PMG, which is a bovine protomorphogen devoid of active thyroxine. She sought to improve her dietary patterns, improve energy, cognition, resilience, and become more educated about dietary and lifestyle choices that could support her health. She entered the program with a severe symptom burden (MSQ = 106) with the worst symptoms affecting the HEENT, dermatological, cardiac, respiratory, gastrointestinal, genitourinary, musculoskeletal, neuropsychological and immune systems. More specifically, her complaints consisted of headaches, blurred/tunnel vision, excessive mucus, canker sores, acne, dry skin, rashes, hair loss, flushing, palpitations, shortness of breath/difficulty taking a deep breath, constipation, bloating, abdominal pain, joint pain, stiffness, muscle aches, fatigue, tiredness, lethargy, poor memory and concentration, poor coordination, indecisiveness, anxiety, mood swings, depressed mood, frequent urination and frequent illness. She additionally complained of food cravings and compulsive eating.

Her initial FFQ revealed a largely vegetarian-based diet with infrequent chicken and egg consumption, a large variety of fruits, numerous whole grains, pea protein, coffee and tea with no alcohol consumption. Outside of the previously mentioned dietary supplement, she was taking zinc, selenium, vitamin D, vitamin K2 and cod liver oil.

Initial laboratory findings revealed a TSH of 1.49 μIU/mL with total and free hormone levels within normal ranges. She was noted with initial TPO antibodies of 120 IU/mL, TGA of <0.09 IU/mL, a hs-CRP of 1.06 mg/L, and a low WBC count of 3.3 x 10^3^ / μL.

Initial organic acid nutritional testing revealed no concerning vitamin or mineral deficiencies. Toxic element screen revealed no concerning findings. RBC analysis for RBC PUFA volume showed high normal omega-3 volume with a normal omega-3: omega-6 ratio.

Stool testing no bacterial overgrowth with slightly low SCFAs. There was no evidence of lipid malabsorption.

At week six of the program, the participant was counseled to continue the consumption of omega-3 fatty fish and increase animal protein consumption, including organ meats, and well as fermented foods.

Following the program, the participant’s MSQ significantly decreased from 106 to 23 with the only continued significant symptoms of hair loss (improved), headaches (improved), and hyperhidrosis. She reported significantly increased energy, improved sleep, fewer food cravings, improved resilience, resolved joint pain, decreased depression, less frequent and severe migraines, no significant gastrointestinal symptoms, and marked improvements in dry skin/ acne, and improved cognitive functioning. She reported significantly improved functioning in her job as a healthcare provider.

A review of her second FFQ documenting the 10-week program revealed strict adherence to the AIP diet with the elimination of refined carbohydrates, potatoes, eggs, legumes, nuts, seeds, grains, and dairy, as well as increases in the consumption of vegetables and fruit, and the new regular inclusion of chicken, kombucha, fermented foods, coconut products, such as coconut yogurt, and AIP starches such as cassava.

Repeat laboratory testing revealed a post-intervention TSH of 3.48 μIU/mL, with very slight increases in free and total hormone levels. hs-CRP decreased from 1.06 to 0.16 mg/L. TPO antibodies decreased from 120 to 105 IU/mL, and TGA remained clinically unchanged <0.9 IU/mL. Interestingly, the patient’s previously low WBC count of 3.3 x 10^3^/μL increased at post-intervention to 4.0 x 10^3^/μL. Her monocyte percentage decreased from an elevated 14% to within normal limits at 9% and her lymphocytes increased from 36% pre-intervention to 42% post-intervention.

Repeat nutritional testing revealed overall balanced vitamin and minimal markers with the exception of a now elevated FIGLU, suggesting folate deficiency [23]. She remained with ideal RBC volume of omega-3 fatty acids as well as no concerning levels of whole blood heavy metals. Repeat stool testing revealed no identifiable potentially pathogenic organisms as well as low normal SCFAs. There were still no signs of lipid malabsorption.

Upon completion of the study, the participant remained without the use of thyroid hormone replacement medication.

Case 17

Case 17 involved a 27-year-old female, with a significant past medical history within the past year of a severe varicella zoster infection, slight elevation in anti-CCP antibodies, without clinical evidence of rheumatoid arthritis, and a diagnosis of HT three months prior to study onset, who began the study on a small dose of Armour (15 mg). She sought to improve her dietary patterns, improve energy, cognition, resilience, and become more educated about dietary and lifestyle choices that could support her health. She hoped to alleviate the most troubling symptoms of fatigue, moodiness, hair loss, and dry skin and reverse HT such that she would no longer need replacement medication.

She entered the program with a moderate symptom burden (MSQ = 75), with the worst symptoms affecting the HEENT, skin, gastrointestinal, genitourinary, neuropsychological, and immune parameters. More specifically, her complaints consisted of watery or itchy eyes and swollen red eyelids, stuffy nose, excessive mucus, stuffy nose, sinus problems, canker sores, acne, dry skin, rashes, hair loss, flushing, constipation, bloating, abdominal pain, fatigue, tiredness, lethargy, poor memory and concentration, indecisiveness, anxiety, mood swings, irritability, frequent urination, and frequent illness.

Her initial FFQ revealed a diet transitioning from a vegetarian template to now regular chicken, egg, and fish consumption, non-dairy creamer with no regular dairy consumption, a large variety of fruits, unrefined whole grains, infrequent rice, numerous nuts and seeds, coconut, decaf coffee, occasional tea, with no alcohol consumption. She had discontinued supplementation prior to the study but reported previous use of a B complex, vitamin D, iron, collagen, oregano, quercetin, and magnesium.

Initial laboratory findings revealed a TSH of 1.77 μIU/mL, with total and free hormone levels within normal ranges. Her additional initial labs included TPO antibodies of 138 IU/mL, TGA of 66.6 IU/mL, hs-CRP of 0.88 mg/L, and a borderline low WBC count of 3.5 x 10^3^/μL and 10% monocytes.

Initial organic acid nutritional testing revealed no concerning vitamin deficiencies. Zinc levels were borderline low and significantly lower than plasma copper, resulting in a depressed copper to zinc ratio. Toxic element screen revealed no concerning findings with only a high normal whole blood mercury at 2.64 mcg/L. RBC analysis for RBC PUFA volume showed high normal omega-3 volume with a normal omega-3/6 ratio.

Stool testing revealed numerous imbalances, including mild lipid malabsorption, low butyrate, and an overgrowth of Citrobacter freundii and Morganella morganii.

At week six of the program, the participant was counseled to continue the consumption of omega-3 fatty fish, animal protein, including organ meats, as well as the inclusion of fermented foods. She was provided with education to explore the exclusion of high FODMAP-containing foods, given her symptoms of constipation and bloating [25]. She was additionally supported with information regarding the use of AIP-compliant digestive bitters to support improve the digestion and absorption of nutrients [27].

Following the program, the participant’s MSQ significantly decreased from 75 to 25, with the only continued severe symptoms of hair loss (which had become less frequent and overall improved). She reported significantly increased energy, improved sleep, improved resilience, improved sinus and allergic symptoms, more stable mood, less severe and frequent gastrointestinal symptoms, and improved cognitive functioning. She reported significantly improved functioning in her job as a healthcare provider.

A review of her second FFQ documenting the 10-week program revealed strict adherence to the AIP diet, with the elimination of refined carbohydrates, potatoes, eggs, legumes, nuts, seeds, grains, and dairy, as well as increases in the consumption of unprocessed meat, vegetables, and fruit, and the new, regular inclusion of more animal protein, fermented foods, and AIP starches.

Repeat laboratory testing revealed a post-intervention TSH of 2.2 μIU/mL, with continued stability in free and total hormone levels. hs-CRP decreased from 0.88 to 0.80 mg/L. TPO antibodies increased slightly from 138 to 155 IU/mL with TGA also slightly increasing from 66.6 to 76.9 IU/mL. Interestingly, the patient’s previously borderline low WBC count of 3.5 x 10^3^/μL had only increased slightly to a post-intervention level of 3.6 x 10^3^/μL. Her monocyte percentages remained at 10%.

Repeat nutritional testing revealed balanced vitamin and mineral markers improved minimal markers, with the exception of a now slightly elevated FIGLU, suggesting folate deficiency [23]. She remained with an ideal RBC volume of omega-3 fatty acids as well as no concerning levels of whole blood heavy metals. Interestingly her plasma copper had decreased from high normal ranges and was now in a nearly 1:1 ratio with plasma zinc.

Repeat stool testing revealed a marked improvement and increase in butyrate. She continued with an overgrowth of C. freundii but now had no evidence of M. morganii. Previously noted mild lipid malabsorption had resolved.

Upon completion of the study, the participant remained using only 15 mg of Armour but was going to seek changing medications to 25 mcg of Tirosint, removing the T3 component, with future considerations for titrating off medication entirely.

Raw data tables

Table 7 includes the baseline laboratory data for the 17 participants completing blood chemistry testing pre-intervention.

**Table 7:** Pre-intervention laboratory data including thyroid parameters, thyroid antibodies, WBC, and differential cell count. Note: TGA <0.9 IU/mL was reported as 0 in the table and treated as 0 in the statistical analysis. TPO antibodies >600 IU/mL were treated as 600 IU/mL in the statistical analysis. AIP (autoimmune protocol), TSH (thyroid stimulating hormone), TPO (thyroid peroxidase antibodies), TGA (anti-thyroglobulin antibodies), WBC (white blood cell), hs-CRP (high sensitivity C-reactive protein), (*) acutely sick, (**) decreased or changed thyroid medication during the study because of the pre-intervention result or because of irregular medication dosing, (***) hs-CRP outlier, (****) WBC count outlier, (#) did not complete post-intervention testing

Lab	AIP 001 (*,** )	AIP 002 (***)	AIP 003 (**)	AIP 004	AIP 005 (#)	AIP 006	AIP 007	AIP 008	AIP 009	AIP 010	AIP 011	AIP 012	AIP 013 (****)	AIP 014	AIP 015 (**)	AIP 016	AIP 017
TSH (μIU/mL)	0.424 (*,** )	4.75	3.55 (**)	2.85	1.48	1.64	1.06	13.45	2.79	2.09	4.08	0.736	0.653	0.42	0.026 (**)	1.49	1.77
total T4 (μg/dL)	12.3 (*,** )	6.5	2 (**)	8.3	7.6	8.1	6.5	6.1	4.7	6.5	7.8	7.4	7.6	5.5	6.8 (**)	6.4	8.2
total T3 (ng/dL)	149 (*,** )	106	131 (**)	101	118	82	80	137	84	90	95	98	142	78	138 (**)	97	115
free T4 (ng/dL)	2.9 (*,** )	2.6	0.31 (**)	1.4	1.29	1.48	1.11	1.02	0.82	1.02	1.19	1.41	1.31	1.05	1.07 (**)	1.07	1.26
free T3 (pg/mL)	1.36 (*,** )	0.91	3.3 (**)	2.7	3.3	2.2	2.2	3.1	2.2	2.3	2.6	3	3.4	2.3	3.4 (**)	2.6	2.8
reverse t3 (ng/dL)	28.2 (*)	17.1	<5.0 (**)	24.7	14.7	27.3	15.9	12.9	15.7	13.4	15.9	15.4	16.1	16.5	15.9 (**)	13.2	17.1
TPO (IU/mL)	477 (*)	374	135	180	365	135	273	185	471	>600	16	438	30	99	141	120	138
TGA (IU/mL)	5.7 (*)	0	2	603.5	200.7	0	4.7	1.8	3.4	2.1	0	3.2	826.8	0	21.8	0	66.6
WBC (x10^3^ / μL)	8.3 (*)	7.9	5.8	4.8	4.5	6.9	4.8	9	6	6.8	7.3	4.6	6.4 (****)	4.9	4.8	3.3	3.5
Neutrophils (%)	50 (*)	63	59	57	40	69	57	70	55	58	68	58	49	58	63	47	49
Lymphocytes (%)	39 (*)	28	30	33	50	21	28	21	33	37	21	30	34	32	29	36	39
Monocytes (%)	9 (*)	8	7	6	8	8	8	6	8	5	9	9	12	7	7	14	10
Eosinophils (%)	2 (*)	1	4	3	2	1	6	2	3	0	2	2	4	3	1	2	1
Basophils (%)	0 (*)	0	0	1	0	1	1	1	1	0	0	1	1	0	0	1	1
Absolute Neutrophils (x10^3^ / μL)	4.2 (*)	5	3.4	2.8	1.8	4.8	2.8	6.3	3.4	3.9	4.9	2.6	3.2	2.8	3	1.5	1.7
Absolute Lymphocytes (x10^3^ / μL)	3.2 (*)	2.2	1.7	1.6	2.2	1.4	1.3	1.9	2	2.5	1.6	1.4	2.2	1.6	1.4	1.2	1.4
Absolute Monocytes (x10^3^ / μL)	0.7 (*)	0.6	0.4	0.3	0.4	0.5	0.4	0.5	0.5	0.4	0.6	0.4	0.8	0.3	0.3	0.5	0.4
Absolute Eosinophils (x10^3^ / μL)	0.1 (*)	0.1	0.2	0.1	0.1	0.1	0.3	0.1	0.2	0	0.2	0.1	0.2	0.1	0	0.1	0
Absolute Basophils (x10^3^ / μL)	0	0	0	0	0	0.1	0	0.1	0	0	0	0	0.1	0	0	0	0
hs-CRP (mg/L)	14.07 (*)	6.97 (***)	0.23	2.64	0.65	0.72	0.43	2.59	0.67	2.42	0.98	0.29	1.95	1.71	1.85	1.06	0.88

Table 8 includes baseline HRQL for the 17 participants completing the SF-36 pre-intervention.

**Table 8:** Pre-intervention SF-36 subcale scores SF-36 (36-Item Short Form Health Survey); AIP (autoimmune protocol)

SF 36 Score	AIP 001	AIP 002	AIP 003	AIP 004	AIP 005	AIP 006	AIP 007	AIP 008	AIP 009	AIP 010	AIP 011	AIP 012	AIP 013	AIP 014	AIP 015	AIP 016	AIP 017
SF 36 Physical Functioning	65	80	95	75	100	95	85	80	55	90	85	90	60	95	75	55	15
SF 36 Physical Role Functioning	0	50	100	25	100	0	25	25	0	50	25	100	0	100	100	0	0
SF 36 Emotional Role Functioning	0	33.3	33.3	100	33.3	0	0	33.3	0	33.3	100	100	100	100	66.7	33.3	0
SF 36 Vitality	10	20	25	30	75	40	25	30	5	20	15	25	0	50	10	10	25
SF-36 Mental Health	56	68	44	72	76	52	36	36	60	40	48	40	60	68	72	44	60
SF 36 Social Role Functioning	62.5	62.5	62.5	62.5	75	50	62.5	50	37.5	25	37.5	100	50	87.5	87.5	50	75
SF 36 Bodily Pain	67.5	45	67.5	67.5	90	67.5	47.5	67.5	32.5	32.5	35	80	67.5	77.5	47.5	55	90
SF 36 General Health	20	35	40	45	75	80	40	35	10	10	30	60	25	65	55	40	40

Table 9 includes the baseline clinical symptom burden for the 17 participants completing the MSQ pre-intervention.

**Table 9:** Pre-intervention MSQ scores (subscales and total) MSQ (Medical Symptoms Questionnaire), AIP (autoimmune protocol)

MSQ	AIP 001	AIP 002	AIP 003	AIP 004	AIP 005	AIP 006	AIP 007	AIP 008	AIP 009	AIP 010	AIP 011	AIP 012	AIP 013	AIP 014	AIP 015	AIP 016	AIP 017
MSQ Head Score	9	4	2	4	2	7	5	7	4	9	10	8	2	1	6	5	1
MSQ Eye Score	6	3	4	0	3	0	10	3	8	1	9	4	5	3	3	4	5
MSQ Ear Score	3	2	0	0	0	0	2	2	9	5	7	0	1	4	7	0	0
MSQ Nose Score	16	5	13	0	1	0	13	2	16	3	9	0	1	7	0	2	13
MSQ Mouth/Throat Score	3	5	1	0	0	0	6	1	0	9	5	0	1	1	3	1	4
MSQ Skin Score	9	12	7	7	3	0	11	8	6	9	12	8	7	5	11	12	8
MSQ Heart Score	0	1	0	0	1	2	1	2	3	1	4	1	2	0	0	6	0
MSQ Lung Score	1	1	0	0	0	0	1	1	0	0	4	0	1	0	0	4	0
MSQ Digestive Score	14	3	10	9	6	6	12	11	9	14	9	7	5	11	11	10	8
MSQ Joint/Muslce Score	11	13	4	12	1	13	5	15	16	8	18	20	15	3	15	12	5
MSQ Weight Score	16	20	15	7	15	0	10	16	1	10	8	4	3	10	12	10	1
MSQ Energy/Activity Score	12	7	8	4	3	6	12	9	6	10	10	10	11	2	7	9	9
MSQ Mind Score	12	4	12	5	0	13	14	16	28	2	9	4	12	4	12	14	5
MSQ Emotion Score	11	11	4	3	2	9	8	12	7	8	13	13	10	4	11	11	6
MSQ Other Score	3	7	3	4	0	0	4	3	6	0	5	4	1	0	5	6	10
MSQ Total Score	126	98	83	55	37	56	114	108	119	89	132	83	77	55	103	106	75

Table 10 includes the post-intervention laboratory data for the 16 participants completing blood chemistry testing following the 10-week dietary and lifestyle intervention. Note: Participant AIP 005 did not complete the study and, as such, there is no data presented in the table below.

**Table 10:** Post-intervention laboratory data, including thyroid parameters, thyroid antibodies, WBC, and differential cell count. Note TGA <0.9 IU/mL was reported as 0 in the table and treated as 0 in the statistical analysis. TPO antibodies >600 IU/mL were treated as 600 IU/mL in the statistical analysis. AIP (autoimmune protocol), TSH (thyroid stimulating hormone), TPO (thyroid peroxidase antibodies), TGA (anti-thyroglobulin antibodies), WBC (white blood cell), hs-CRP (high sensitivity C-reactive protein), (*) acutely sick, (**) decreased or changed thyroid medication during the study because of the pre-intervention result or because of irregular medication dosing, (***) hs-CRP outlier, (****) WBC count outlier, (#) did not complete post-intervention testing

Lab	AIP 001 (*,**)	AIP 002 (***)	AIP 003 (**)	AIP 004	AIP 005 (#)	AIP 006	AIP 007	AIP 008 (*)	AIP 009	AIP 010	AIP 011	AIP 012	AIP 013 (****)	AIP 014	AIP 015 (**)	AIP 016	AIP 017
TSH (μIU/mL)	0.92 (*,**)	2.32	7.35 (**)	2.06		1.55	1.13	31.92 (*)	3.06	0.535	2.15	0.23	5.07	0.069	0.244 (**)	3.48	2.2
total T4 (μg/dL)	13.1 (*,**)	6.1	5 (**)	9.8		8.3	6.6	8.3 (*)	5.2	6.9	8.8	8	7.1	5.4	6.4 (**)	6.6	6.4
total T3 (ng/dL)	98 (*,**)	93	107 (**)	84		78	84	154 (*)	83	79	78	99	92	101	88 (**)	96	101
free T4 (ng/dL)	1.8 (*,**)	2.3	0.77 (**)	1.59		1.49	1.19	1.1 (*)	0.86	1.23	1.47	1.53	1.14	1.07	0.91 (**)	1.11	1.21
free T3 (pg/mL)	1.61 (*,**)	0.91	2.5 (**)	2.6		2.2	2.5	3. 1(*)	2.3	2.4	2.4	2.8	2.4	2.9	2.2 (**)	2.8	2.8
reverse t3 (ng/dL)	54.7 (*,**)	15.9	10.9 (**)	30.5		27.7	17.9	17.5 (*)	13	14.8	23.3	20.6	18	16.3	20.3 (**)	13.6	17.2
TPO (IU/mL)	598 (*)	409	107	177		160	190	123 (*)	481	>600	14	452	29	75	111	105	155
TGA (IU/mL)	3.1 (*)	0	0.9	735.7		0	4.8	4.5 (*)	3.6	1.7	0	4.8	884.3	0	20.7	0	76.9
WBC (x10^3^ / μL)	6.1(*)	6.6	4.4	3.8		5.9	3.9	11.1 (*)	5.5	4.6	6.7	4.5	8.3 (****)	4.2	5.6	4	3.6
Neutrophils (%)	58 (*)	66	57	51		59	55	76 (*)	44	48	71	57	52	61	66	44	51
Lymphocytes (%)	32 (*)	28	31	38		32	34	17 (*)	44	46	19	32	37	29	27	42	37
Monocytes (%)	10 (*)	5	8	7		7	6	5 (*)	7	6	8	9	7	7	7	9	10
Eosinophils (%)	0 (*)	1	4	3		1	4	1 (*)	4	0	2	2	2	3	0	3	1
Basophils (%)	0 (*)	0	0	1		1	1	1 (*)	1	0	0	0	1	0	0	2	1
Absolute Neutrophils (x10^3^ / μL)	3.5 (*)	4.3	2.5	2		3.5	2.1	8.4 (*)	2.4	2.2	4.7	2.5	4.4	2.6	3.6	1.8	1.8
Absolute Lymphocytes (x10^3^ / μL)	1.9 (*)	1.9	1.4	1.5		1.9	1.3	1.9 (*)	2.4	2.1	1.2	1.4	3.1	1.2	1.5	1.7	1.3
Absolute Monocytes (x10^3^ / μL)	0.6 (*)	0.3	0.4	0.3		0.4	0.2	0.6 (*)	0.4	0.3	0.6	0.4	0.6	0.3	0.4	0.4	0.4
Absolute Eosinophils (x10^3^ / μL)	0 (*)	0.1	0.2	0.1		0	0.2	0.1 (*)	0.2	0	0.1	0.1	0.2	0.1	0	0.1	0
Absolute Basophils (x10^3^ / μL)	0 (*)	0	0	0		0	0	0.1 (*)	0	0	0	0	0.1	0	0	0.1	0
hs-CRP (mg/L)	11.7 (*)	5.02 (***)	0.42	2.9		0.76	0.38	6.52 (*)	0.36	0.84	0.9	0.32	1.63	0.7	0.94	0.16	0.8

Table 11 includes post-intervention HRQL for the 16 participants completing the SF-36 survey following the 10-week dietary intervention. Note: Participant AIP 005 did not complete the study and, as such, there is no data presented in the table below.

**Table 11:** Post-intervention SF-36 subscale scores AIP (autoimmune protocol), SF-36 (36 Item Short Form Health Survey), # (did not complete post-intervention testing)

SF 36 Score	AIP 001	AIP 002	AIP 003	AIP 004	AIP 005 (#)	AIP 006	AIP 007	AIP 008	AIP 009	AIP 010	AIP 011	AIP 012	AIP 013	AIP 014	AIP 015	AIP 016	AIP 017
SF 36 Physical Functioning	70	95	100	90		95	95	95	95	100	95	100	75	95	80	100	100
SF 36 Physical Role Functioning	100	100	100	100		75	50	25	50	100	100	100	25	100	100	50	100
SF 36 Emotional Role Functioning	33.3	100	100	100		66.7	100	0	100	100	100	100	100	100	0	100	0
SF 36 Vitality	30	70	40	65		70	45	35	35	85	70	75	15	80	55	55	60
SF-36 Mental Health	72	88	76	88		80	60	52	84	100	84	68	68	92	76	64	80
SF 36 Social Role Functioning	87.5	87.5	50	75		100	75	50	75	100	100	87.5	75	100	75	87.5	75
SF 36 Bodily Pain	77.5	77.5	100	90		35	90	75	77.5	100	90	90	67.5	100	67.5	77.5	67.5
SF 36 General Health	50	65	85	55		95	60	35	40	90	75	85	50	85	70	80	70

Table 12 includes the post-intervention clinical symptom burden for the 16 participants completing the MSQ following the 10-week dietary intervention. Note: Participant AIP 005 did not complete the study and, as such, there is no data presented in the table below.

**Table 12:** MSQ subscale and total scores post-intervention AIP (autoimmune protocol), MSQ (Medical Symptoms Questionnaire), (#) did not complete post-intervention testing

MSQ	AIP 001	AIP 002	AIP 003	AIP 004	AIP 005 (#)	AIP 006	AIP 007	AIP 008	AIP 009	AIP 010	AIP 011	AIP 012	AIP 013	AIP 014	AIP 015	AIP 016	AIP 017
MSQ Head Score	6	0	1	2		2	6	9	1	0	0	0	0	0	1	2	0
MSQ Eye Score	2	3	2	0		1	7	6	3	0	0	0	3	0	3	2	2
MSQ Ear Score	0	0	0	0		0	0	0	0	1	0	0	1	1	0	0	0
MSQ Nose Score	11	1	0	0		0	7	1	1	0	0	0	0	1	1	0	5
MSQ Mouth/Throat Score	2	0	0	0		0	2	1	0	1	0	0	0	0	2	1	0
MSQ Skin Score	4	0	1	5		0	5	9	5	3	1	2	3	1	4	7	3
MSQ Heart Score	1	0	0	1		2	0	4	0	0	0	0	0	0	0	2	0
MSQ Lung Score	0	0	0	1		0	0	0	0	0	0	0	0	0	0	0	0
MSQ Digestive Score	4	1	1	3		9	9	11	6	0	2	3	4	2	5	3	4
MSQ Joint/Muslce Score	1	3	0	4		4	1	9	2	0	2	4	5	1	4	0	4
MSQ Weight Score	3	2	2	4		4	4	9	1	0	1	3	1	3	4	4	1
MSQ Energy/Activity Score	4	0	0	2		0	3	8	2	0	1	4	2	1	2	0	1
MSQ Mind Score	1	1	2	2		6	7	8	5	1	0	1	3	1	8	1	0
MSQ Emotion Score	3	0	0	3		4	4	7	4	0	1	8	3	1	2	1	3
MSQ Other Score	1	1	1	1		1	1	3	2	0	0	0	0	0	0	0	2
MSQ Total Score	43	12	10	28		33	56	85	32	6	8	25	25	12	36	23	25

Table 13 depicts the originally calculated p-values, ordered from lowest to highest matched with corresponding corrected p-values for statistical significance following the use of a false discovery rate correction, given the study's multiple hypotheses. The corrected p-values of significance for n = 27 tests were calculated assuming a false discovery rate d = 0.05 using the formula p_i _= d * (i/n), where n is the number of tests, i is an integer between 1-27, and p_i _is the corrected p-value for the given ordered integer. After performing the correction and matching the ordered and previously calculated p values with its respective p_i_, the only original p-value affected corresponded to a change in the mean lymphocyte count from pre- to post-intervention. Given this correction, the study authors could not reliably state that there was a significant difference between the mean lymphocyte count from pre- to post-intervention. No other p-values were affected by the false discovery rate correction.

**Table 13:** False discovery rate corrections for p-values i (integer: 1-27), P_i_ value (corrected p-value), * (correction to not statistically significant)

	P1	P2	P3	P4	P5	P6	P7	P8	P9	P10	P11	P12	P13	P14	P15	P16	P17	P18	P19	P20	P21	P22	P23	P24	P25	P26	P27
i	1	2	3	4	5	6	7	8	9	10	11	12	13	14	15	16	17	18	19	20	21	22	23	24	25	26	27
P_i_ value	0.0019	0.0037	0.0056	0.0074	0.0093	0.0111	0.0130	0.0148	0.0167	0.0185	0.0204	0.0222	0.0241	0.0259	0.0278*	0.0296	0.0315	0.0333	0.0352	0.0370	0.0389	0.0407	0.0426	0.0444	0.0463	0.0481	0.0500
Original p-value	0.0001	0.0001	0.0001	0.0001	0.0001	0.0010	0.0020	0.0020	0.0057	0.0063	0.0110	0.0110	0.0112	0.0219	0.0286*	0.0684	0.0743	0.1240	0.1396	0.176	0.183	0.385	0.418	0.455	0.565	0.882	0.942
